# Early Low-Titer Neutralizing Antibodies Impede HIV-1 Replication and Select for Virus Escape

**DOI:** 10.1371/journal.ppat.1002721

**Published:** 2012-05-31

**Authors:** Katharine J. Bar, Chun-yen Tsao, Shilpa S. Iyer, Julie M. Decker, Yongping Yang, Mattia Bonsignori, Xi Chen, Kwan-Ki Hwang, David C. Montefiori, Hua-Xin Liao, Peter Hraber, William Fischer, Hui Li, Shuyi Wang, Sarah Sterrett, Brandon F. Keele, Vitaly V. Ganusov, Alan S. Perelson, Bette T. Korber, Ivelin Georgiev, Jason S. McLellan, Jeffrey W. Pavlicek, Feng Gao, Barton F. Haynes, Beatrice H. Hahn, Peter D. Kwong, George M. Shaw

**Affiliations:** 1 Perelman School of Medicine, University of Pennsylvania, Philadelphia, Pennsylvania, United States of America; 2 Duke University School of Medicine, Durham, North Carolina, United States of America; 3 University of Alabama at Birmingham, Birmingham, Alabama, United States of America; 4 Vaccine Research Center, National Institute of Allergy and Infectious Disease, National Institutes of Health, Bethesda, Maryland, United States of America; 5 Los Alamos National Laboratory, Los Alamos, New Mexico, United States of America; 6 SAIC-Frederick Inc, National Cancer Institute, Frederick, Maryland, United States of America; 7 University of Tennessee, Knoxville, Tennessee, United States of America; University of Zurich, Switzerland

## Abstract

Single genome sequencing of early HIV-1 genomes provides a sensitive, dynamic assessment of virus evolution and insight into the earliest anti-viral immune responses *in vivo*. By using this approach, together with deep sequencing, site-directed mutagenesis, antibody adsorptions and virus-entry assays, we found evidence in three subjects of neutralizing antibody (Nab) responses as early as 2 weeks post-seroconversion, with Nab titers as low as 1∶20 to 1∶50 (IC_50_) selecting for virus escape. In each of the subjects, Nabs targeted different regions of the HIV-1 envelope (Env) in a strain-specific, conformationally sensitive manner. In subject CH40, virus escape was first mediated by mutations in the V1 region of the Env, followed by V3. HIV-1 specific monoclonal antibodies from this subject mapped to an immunodominant region at the base of V3 and exhibited neutralizing patterns indistinguishable from polyclonal antibody responses, indicating V1–V3 interactions within the Env trimer. In subject CH77, escape mutations mapped to the V2 region of Env, several of which selected for alterations of glycosylation. And in subject CH58, escape mutations mapped to the Env outer domain. In all three subjects, initial Nab recognition was followed by sequential rounds of virus escape and Nab elicitation, with Nab escape variants exhibiting variable costs to replication fitness. Although delayed in comparison with autologous CD8 T-cell responses, our findings show that Nabs appear earlier in HIV-1 infection than previously recognized, target diverse sites on HIV-1 Env, and impede virus replication at surprisingly low titers. The unexpected *in vivo* sensitivity of early transmitted/founder virus to Nabs raises the possibility that similarly low concentrations of vaccine-induced Nabs could impair virus acquisition in natural HIV-1 transmission, where the risk of infection is low and the number of viruses responsible for transmission and productive clinical infection is typically one.

## Author Summary

Characterizing early adaptive immune responses to HIV-1 can inform studies of virus persistence, pathogenesis and natural history and can guide rational vaccine design. Previous studies examined the role of neutralizing antibodies (Nab) in acute and chronic HIV-1 infection but not against the precise envelope (Env) glycoproteins of transmitted/founder (T/F) viruses and not in direct comparison with autologous cellular immune responses in the same subjects. Here, we identified T/F HIV-1 *env* genes and their progeny in three subjects by single genome sequencing and performed a dynamic assessment of Nab responses based on *env* evolution and phenotypic changes in the Env glycoprotein over time. Surprisingly, we found genetic evidence of Nab activity as early as 2 weeks post-seroconversion, with Nab titers as low as 1∶20 to 1∶50 (IC_50_) selecting for virus escape. Nabs targeted different regions of the HIV-1 envelope (Env) in a strain-specific, conformationally sensitive manner. Although delayed in comparison with autologous CD8 T-cell responses, Nabs appeared earlier in HIV-1 infection than previously recognized and impeded virus entry at low titers. This raises the possibility that similarly low concentrations of vaccine-induced Nabs could impair virus acquisition in natural HIV-1 transmission, where the risk of infection is low and the number of viruses responsible for transmission and productive clinical infection is typically one.

## Introduction

Much of what is known about virus-host interactions underlying HIV-1 persistence and pathogenesis in humans has come from quantitative measurements and mathematical modeling of viral replication dynamics and virus evolution in response to selective pressures, including antiretroviral drug therapy and adaptive immune responses [1]–[9]. Comparable insights into HIV-1 transmission have been gleaned from analyses of acute infection viral sequences derived by single genome amplification (SGA) and interpreted in the context of a model of random virus evolution [8], [10], [11]. This latter approach makes possible an unambiguous molecular identification of actual transmitted/founder (T/F) viruses that are responsible for establishing productive clinical infection by HIV-1 in humans [8], [10], [12]–[15] and by SIV in rhesus macaques [16]–[18]. Importantly, because the SGA - direct amplicon sequencing strategy precludes *Taq*-polymerase mediated recombination and nucleotide misincorporation errors in finished sequences, it allows for the analysis of mutational linkage across complete viral genes and genomes [8], [10]. Based on these considerations, we postulated that a precise molecular identification of T/F virus genomes and their evolving progeny could enable a comprehensive proteome-wide assessment of the earliest adaptive immune responses that shape and constrain the early replicating HIV-1 quasispecies. This hypothesis was affirmed for HIV-specific cytotoxic T-cell (CTL) responses in three acutely-infected subjects [7], [8]. Here, we examined in the same three subjects whether this strategy could illuminate the earliest virus-specific neutralizing antibody (Nab) responses.

Nabs constrain the replication of most viruses and are essential to the efficacy of most viral vaccines [19], and this is presumed to be the case for HIV-1 [19]–[22]. There is clear evidence in Indian rhesus macaque models of SHIV infection that Nabs directed toward HIV-1 gp41 or gp120 can confer sterilizing immunity [23]–[25]. Further evidence in support of the protective potential of Nabs has come from heterologous low-dose mucosal SIV infection in Env-vaccinated rhesus macaques [26]. In humans, however, antibody correlates of protection from infection are still being identified [27]–[29] and the minimum titers of Nabs necessary to impede virus infection *in vivo* have not been determined, although it is clear that moderate and high titers of Nabs can lead to HIV-1 selection and escape [6], [30]–[36]. The present study thus focused on four aspects of the Nab response in HIV-1 infected humans: (i) identification of genetic ‘footprints’ of the earliest detectable Nab responses to HIV-1; (ii) characterization of Env epitopes recognized by the earliest Nabs and molecular pathways of virus escape; (iii) determination of the titers of Nabs that are sufficient to select for virus escape *in vivo*; and (iv) viral replication fitness costs associated with Nab escape.

Previous studies have addressed some of these same questions but with different experimental strategies that allowed for lesser degrees of molecular and dynamic resolution. Wei [6] and Richman [30] first used single-cycle Env *trans*-complementation assays to detect autologous strain-specific Nab responses, but neither study used SGA to identify T/F viruses or to look for genetic linkage of mutational escape pathways, nor did they use deep sequencing methods to detect the earliest escape mutations. Other investigators examined Nab responses against early viruses but without deep sequencing or a detailed kinetic analysis of low-titer antibody effects [32]–[37]. Here, we hypothesized that an in-depth kinetic analysis of the evolution of T/F viral *env* genes could provide for the most sensitive detection of Nab pressure on the replicating virus quasispecies — even before direct phenotypic detection of virus neutralization *in vitro* — and that such findings could be corroborated and extended by deep sequencing, site-directed mutagenesis, antibody adsorptions and *in vitro* testing of T/F and escape variant Env proteins for neutralization by autologous antibodies.

## Results

### Neutralization of Transmitted/Founder viruses

Subjects CH40, CH77 and CH58 were each productively infected by single T/F viruses as demonstrated by SGA or 454 deep sequencing [8], [38]. These T/F viruses exhibited a phenotype typical of primary HIV-1 strains including CD4 dependence, CCR5 tropism (CH40 and CH58) or CCR5/CXCR4 dual tropism (CH77), resistance to CD4-induced and V3-specific antibodies, variable sensitivity to the broadly neutralizing antibodies b12, 4E10, 2F5 and HIVIG, and CD4+ T tropism [8], [10], [39]. We tested the three T/F viruses for neutralization sensitivity to autologous and heterologous plasma antibodies. Autologous neutralization titers (IC_50_) at 3, 6 and 12 month time points were 1∶1446/1∶2432/1∶1282 (CH40), 1∶38/1∶100/1∶239 (CH77) and <1∶20/1∶48/1∶243 (CH58) (Figure S1; Table 1). None of the plasma specimens exhibited heterologous neutralizing activity at dilutions as low as 1∶20. The kinetics of appearance and magnitude of autologous Nab responses and the corresponding plasma viral load and CD4+ T cell measurements (Figure S1), were typical of HIV-1 subtype B infections [6], [30], [40].

**Table 1:** Neutralization sensitivities of Env clones and site-directed mutants to autologous plasma and monoclonal antibodies.

	Env clone/mutant	day 45 plasma^a^	day 111 plasma	day 181 plasma	AbCH83^b^	AbCH84
CH40	T/F Env	<20	1446 (±330)	2432 (±900)	0.076 (±.012)	0.034 (±.0067)
	E146K	<20	38 (±30)*	3288 (±1237)	>10*	>10*
	E146G	<20	69 (±42)*	1718 (±397)	>10*	>10*
	G145E	<20	523 (±99)*	1146 (±305)	0.13 (±.02)*	0.038 (±.00076)
	N144K	<20	736 (±347)	1507 (±441)	0.056 (±.008)*	0.046 (±.0046)
	N139T/E146T/M147L	<20	37 (±11)*	186 (±14)*	>10*	>10*
	E146G/R327K/E332K	<20	18 (±10)*	39 (±35)*	>10*	>10*
	R327K/E332K	<20	20 (±14)*	90 (±66)*	>10*	>10*
	R327K	<20	529 (±49)*	1216 (±247)	0.038 (±.011)*	0.018 (±.0016)*
	E332K	<20	532 (±62)*	1098 (±87)	0.033 (±.014)*	0.018 (±.0015)*
	T295N	<20	332 (±102)*	471 (±135)*	0.17 (±.030)*	.076 (±.012)*
	N300K	<20	24 (±22)*	248 (±148)*	>10*	>10*
	K160N	<20	1575 (±596)	2048 (±666)	0.080 (±.0073)	0.029 (±.0061)

		day 32 plasma	day 102 plasma	day 159 plasma
CH77	T/F Env	<20	38 (±4.7)	100 (±66)
	N130D	<20	22 (±12)	85 (±39)
	T186A	<20	17 (±6.1)*	45 (±5.8)
	K187E	<20	10 (±0)*	32 (±6.7)
	T187aN	<20	17 (±6.4)*	39 (±1.0)
	T187aA	<20	14 (±6.9)*	44 (±4.6)
	N187bS	<20	17 (±6.1)*	64 (±6.0)
	N187bD	<20	14 (±6.9)*	71 (±8.2)
	T188I	<20	10 (±0)*	41 (±10)
	del 187b–188	<20	20 (±8.9)*	45 (±11)
	D230E	<20	27 (±5.5)	68 (±17)

		day 85 plasma	day 154 plasma	day 350 plasma
CH58	T/F Env	<20	48 (±1.5)	243 (±119)
	T232A	<20	48 (±11)	172 (±57)
	E336K	<20	23 (±12)*	122 (±5.5)
	N339H	<20	22 (±10)*	110 (±53)
	N339T	<20	31 (±24)	156 (±77)
	N339S	<20	30 (±18)	92 (±41)
	N339D	<20	21 (±16)*	88 (±5.7)
	K343Q	<20	28 (±16)	136 (±71)
	K343R	<20	29 (±17)	158 (±49)
	G410E	<20	20 (±8.7)*	53 (±6.9)*
	del 409–411	<20	19 (±8.2)*	36 (±4.2)*

a Neutralization sensitivity determined in TZM assay using autologous plasma starting at 1∶20 dilution and reported as the IC_50_ titer. Each value represents the mean (± standard deviation) of at least three independent experiments. For calculating means and standard deviations, IC_50_<20 were counted as 10.

*: p<0.05, when mutant titers were compared with the T/F titer by two-tailed t-test.

b Neutralization sensitivity to autologous monoclonal antibodies (AbCH83 and AbCH84) from day 132 B-cells starting at a concentration of 10 ug/ml and reported in ug/ml IC_50_. Each value is the mean of at least three independent experiments.

### Neutralizing antibody escape by 6 month viruses and costs to viral replication fitness

**Figure 1:**
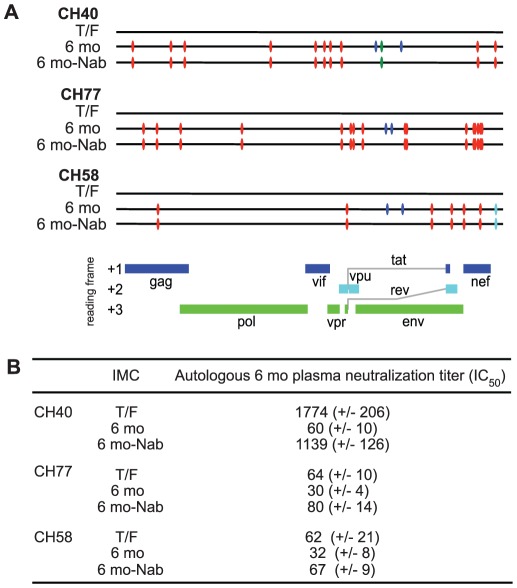
Sequences and autologous neutralization sensitivities of consensus infectious molecular clones. A. 6 mo and 6 mo-Nab IMC sequences are aligned to the T/F sequence with red and blue tics indicating non-synonymous changes implicated in CTL and Nab escape, respectively. Green tics denote synonymous changes and aqua tics changes in non-coding regions. B. Neutralization of IMCs by autologous 6 month plasma is reported as mean (+/− SD) reciprocal plasma dilutions (IC_50_). Experiments were conducted in triplicate and repeated three times.

**Figure 2:**
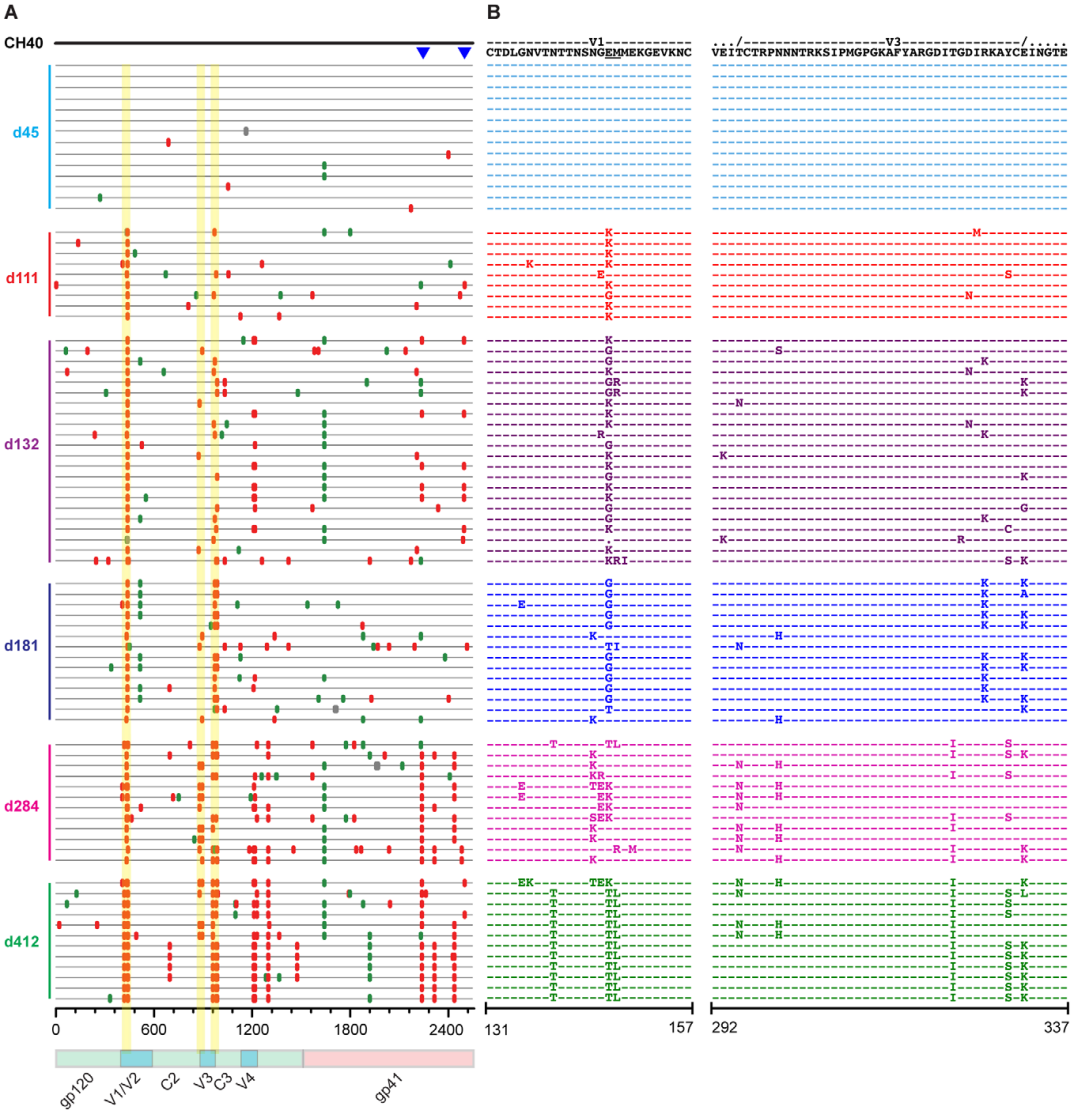
*Highlighter* analysis and *env* sequence alignments of putative Nab epitopes in subject CH40. A. *Highlighter* plot traces acquired mutations in gp160 *env* against the T/F sequence at top. Nucleotide differences from the T/F sequence are indicated (red: non-synonymous, green: synonymous) according to days post-seroconversion. CTL epitopes previously confirmed in T cell assays, are indicated by blue triangles. Mutations responsible for Nab escape are highlighted in yellow. B. Amino acid alignments of the V1 and V3 regions (HXB2 numbering). The two amino acid span interrogated by PASS is underlined. SGA sequences were from 6 independent experiments.

In each subject, we examined the neutralization sensitivity of full-length infectious molecular clones (IMCs) of the T/F virus compared with IMCs of consensus 6 month sequences (Figure 1). The latter IMCs, like the T/F viruses, were all replication competent (Figure S2) and contained phenotypically confirmed CTL escape mutations in addition to putative Nab escape mutations (Figures 1A, 2–4). We found neutralization of the T/F IMCs by 6 month plasma antibodies in titers comparable to those detected using Env pseudotyped viruses (Table 1; Figure S1). 6 month consensus IMCs containing putative Nab escape mutations in Env showed significant resistance to neutralization compared with T/F viruses and 6 month IMCs lacking putative Nab escape mutations (p<0.05 for each, two-tailed paired t-test) (Figure 1B). Replication fitness costs resulting from Nab escape were analyzed in competitive replication assays by comparing T/F IMCs to 6 month IMCs with and without Nab escape mutations. These analyses suggested minimum costs to viral fitness from Nab escape mutations of between 0% and 24% (Figure S2 and Dataset S1).

### Single genome sequencing reveals early selection at putative Nab epitopes

**Figure 3 ppat-1002721-g003:**
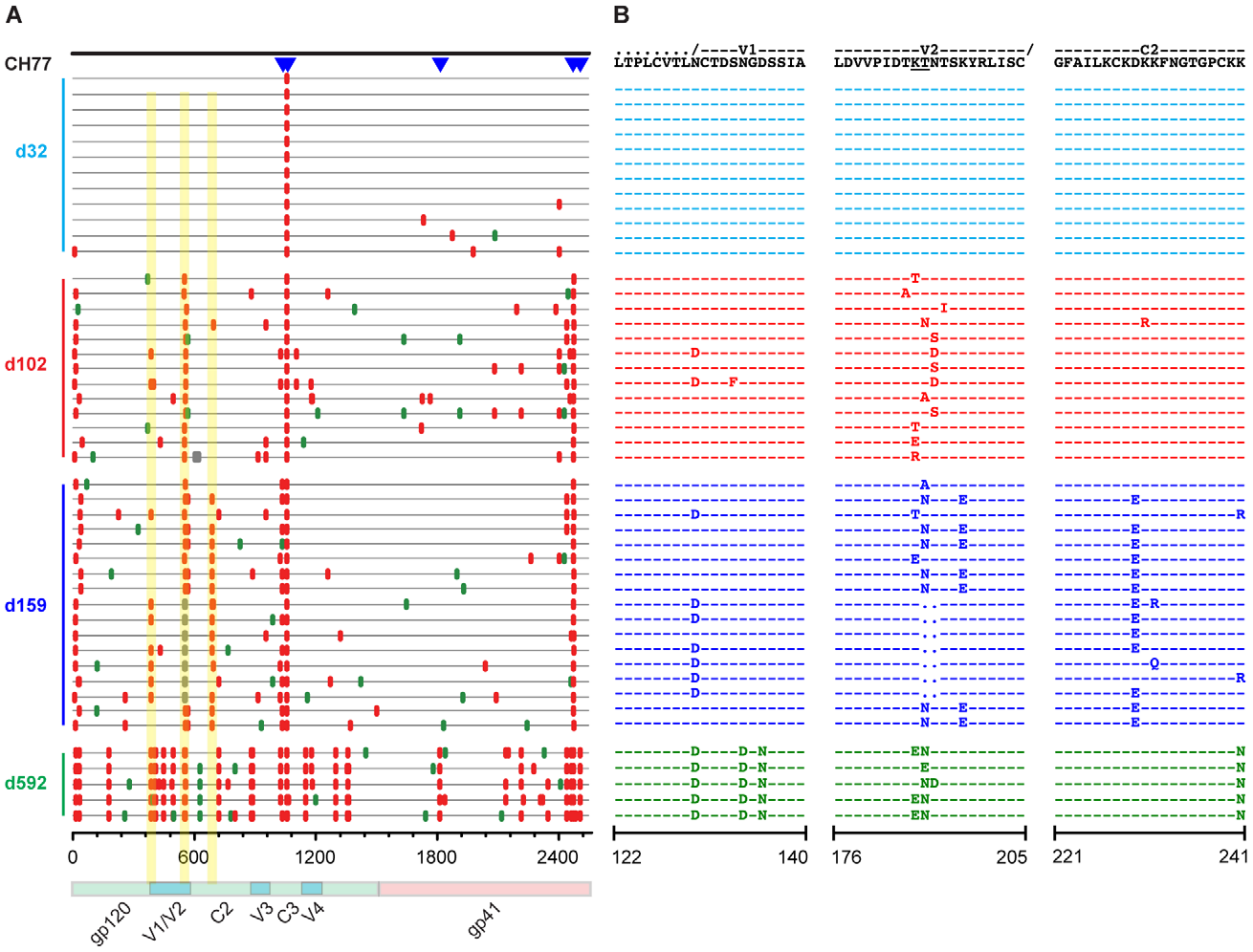
*Highlighter* analysis and *env* sequence alignments of putative Nab epitopes in subject CH77. A. *Highlighter* plots as described in Figure 2. B. Amino acid alignments of segments of V1, V2 and C2 regions as described in Figure 2. SGA sequences were obtained from 7 independent experiments.

**Figure 4:**
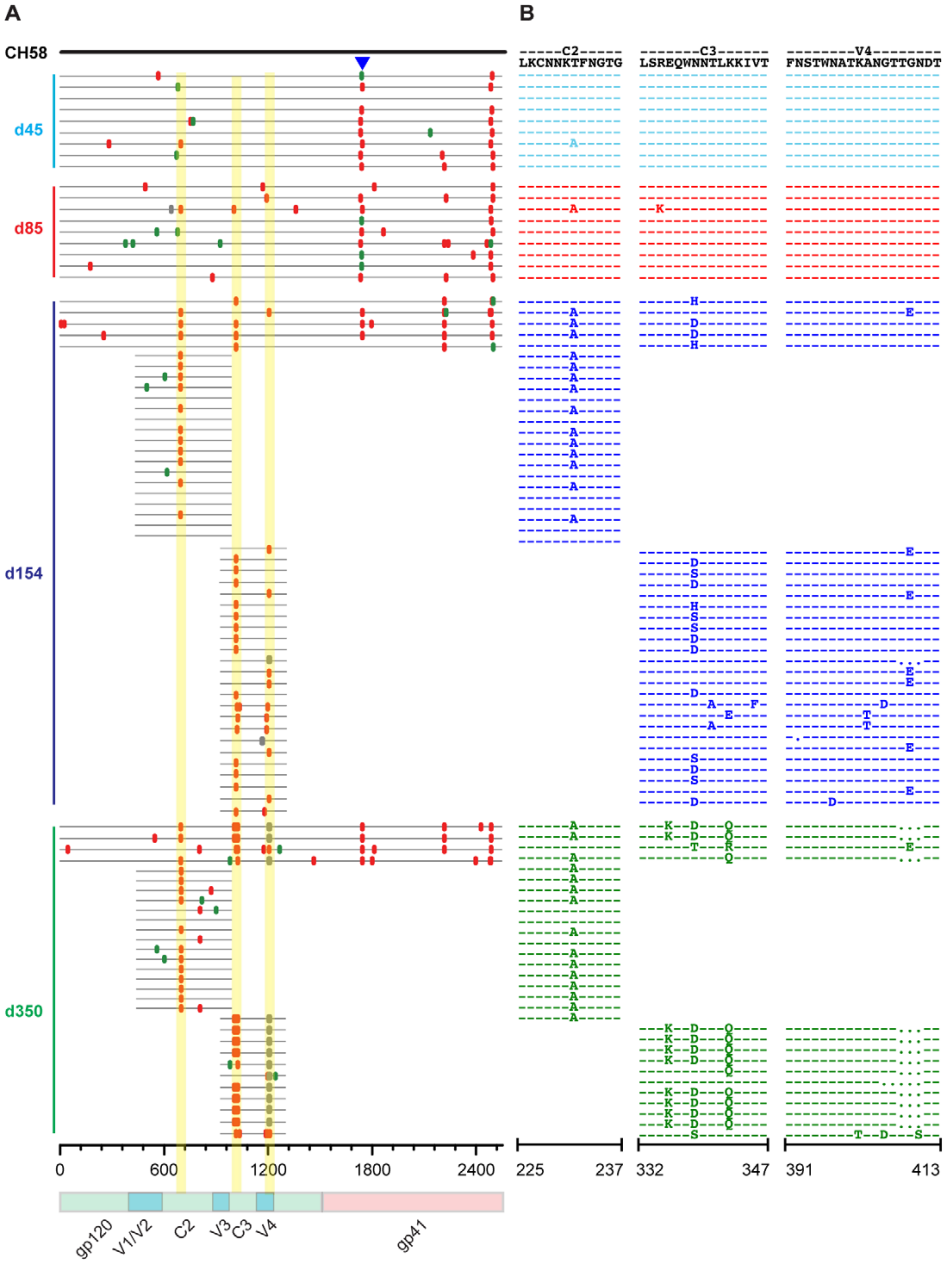
*Highlighter* analysis and *env* sequence alignments of putative Nab epitopes in subject CH58. A. *Highlighter* plots as described in Figure 2. B. Amino acid alignments of segments of the C2, C3, and V4 regions as described in Figure 2. SGA sequences were obtained from 12 independent experiments.

SGA sequencing of sequential plasma specimens was used to further characterize candidate Nab epitopes by looking for the earliest indications of Nab selection and escape across full-length gp160 *env* sequences. Figures 2–4 depict the temporal accumulation of nonsynonomous and synonomous *env* mutations in subjects CH40 (Figure 2A), CH77 (Figure 3A), and CH58 (Figure 4A).

#### CH40

For subject CH40, 83 SGA-derived *env* sequences from longitudinal time points 45–412 days post-seroconversion were aligned beneath the T/F *env* sequence (Figure 2A). The median number of *env* sequences per time point was 12 (range 9–22). Sequences from the earliest sample 45 days post-seroconversion were highly homogeneous (0.04% maximal diversity) and conformed to a Poisson distribution of random changes and “star-like” phylogeny [10], [11]. In contrast, by day 111, there was evidence of complete replacement of the T/F virus with a population of mutants that differed in one of two adjacent amino acids at positions 145 and 146 in V1 (Figure 2B). Three different substitutions combined to completely replace the T/F sequence over this two amino acid span. Eight of nine sequences contained a substitution at position 146, including seven of nine sequences with an identical E146K substitution. Later time points confirmed persistent selection pressure directed against this narrow span of V1, with the virus exploring multiple amino acid substitutions within this region; glutamate was the predominate amino acid at transmission, which was replaced by a lysine at day 111 (7/9 sequences), a glycine by day 181 (10/14 sequences), and a threonine by day 412 (11/12 sequences). The selection pressure in this region also altered potential N-linked glycosylation (PNLG) sites, with the first alteration seen in two sequences at day 181 and subsequent emergence and fixation of a sequence motif (N139T/E146T/M147L) that resulted in a shift of a PNLG site from position 139 to 144 at day 412.

In addition to changes in V1, selected mutations emerged early in the N- and C-terminal regions of V3 (Figure 2A and 2B). These mutations were non-randomly distributed as assessed by formal statistical analyses previously reported [7]. At day 111, only 3 of 9 sequences contained changes in the C-terminal V3 base. By day 132, 15 of 22 sequences had polymorphisms in either or both of the N- or C-termini of V3. By day 181, mutants had completely replaced the T/F sequence at the V3 base (N- or C-termini), and this replacement persisted through one year of infection. The N-terminal V3 mutations were associated with the addition of a PNLG site at position 300. The selection pressure at the N- and C-termini of V3 appeared to be interrelated, with sequences containing changes in one area or the other, but rarely both. This, along with the structural contiguity of the N- and C-terminal V3 base, suggested that these two discontinuous polymorphic sites might comprise a single epitope such that mutations in either site could confer virus escape from a monotypic Nab response, a hypothesis that we subsequently validated (see below). Late selection at two positions in the carboxyterminus of gp41 represented CTL escape mutations [7].

#### CH77

Sequential gp160 *env* sequence analyses were also performed on CH77 (days 32–592) and CH58 (days 45–350). For CH77, 47 SGA *env* sequences (median 15; range 5–17) were obtained at four time points. Previously, Goonetilleke [7] phenotypically confirmed five CTL epitopes in the CH77 gp160 Env, plus an additional site of selection in the signal peptide predicted to be a CTL epitope (Figure 3A). Excluding these changes, we identified shared mutations in V1, V2 and C2 that replaced the T/F sequence in the majority of sequences within the first six months of infection. These clustered mutations were located in regions previously associated with Nab escape [41] and either directly involved or flanked PNLG sites, which further suggested their potential involvement in Nab recognition (Figure 3B). By day 102, the V2 region had accumulated nine different amino acid polymorphisms that caused complete replacement of the T/F sequence. The V2 mutations abutted a PNLG site in the T/F sequence and 46% of these day 102 substitutions altered the glycan site. Perturbations of potential glycans increased over time, with ≥80% of later sequences containing mutations altering a PNLG. The next potential Nab epitope was in C2, where three distinct polymorphisms over a 12 amino acid span led to complete replacement of the T/F sequence by day 159 (Figure 3B). Finally, the V1 region also contained evidence of selection; a shared polymorphism that affected a PNLG arose as a minor variant at day 102 and fully replaced the T/F sequence by day 350 post seroconversion. These findings suggested that the evolving virus quasispecies in CH77 contained Nab escape mutations in V1, V2 and C2.

#### CH58

Subject CH58 was a virus controller, with plasma viral loads 6–12 months after infection of 140 to 210 RNA/ml (Figure S1). Due to technical limitations of amplifying plasma virus at lower viral loads, we obtained fewer SGA-derived complete *env* gp160 sequences (27 total, median 7 per time point, range 4–9). By targeting SGA on shorter *env* fragments, we were able to obtain a robust set of sequences (n = 95) for analysis of viral *env* quasispecies evolution (Figure 4A). A confirmed CTL epitope and two areas of selection in the intracytoplasmic domain of gp41 were excluded (Figure 4A). The first evidence of potential Nab escape in gp120 was at day 45 and involved C2. Here, a nucleotide polymorphism conferring a non-synonymous amino acid change that eliminated a PNLG site was evident in one sequence in both day 45 and day 85 samples and became predominant thereafter. In C3, polymorphisms over an eight amino acid span first appeared by day 85 and became predominant by day 154. All but one of the sequences contained mutations that affected a PNLG. Other mutations potentially related to early Nab in CH58 arose in the glycan rich V4 region. Two V4 permutations, a single nucleotide substitution and a three amino acid deletion, arose by day 154 and fully replaced the T/F virus by day 350. The V4 substitution abutted a PNLG site and was the more prevalent of the two mutations at day 154, while the three amino acid deletion abrogated the glycan and represented the majority of sequences at day 350. These findings suggested that the evolving virus quasispecies in CH58 contained Nab escape mutations in C2, C3 and V4.

### Neutralization resistance of early HIV-1 variants

For each subject, we determined the effect on neutralization sensitivity of early amino acid substitutions represented in sequences from the earliest sampled time points through 6 months post-seroconversion (Figures 2–4). This was done using site-directed mutagenesis to introduce mutations alone or in combination into T/F *envs* and testing the Envs for neutralization sensitivity. A total of 31 mutants were tested (Table 1).

#### CH40

Autologous plasma Nabs against the T/F virus were first detected for subject CH40 111 days after seroconversion with titers of 1∶1400 (Figure S1; Table 1). This coincided with the first appearance of selection in V1 by SGA sequencing (Figure 2). An Env mutant containing a single amino acid substitution in V1, E146K, conferred virtually complete escape from day 111 plasma Nab (IC_50_ = 1∶38). A different V1 substitution at the same position, E146G, which was present at low frequency at day 111 but later expanded to become the majority sequence at days 132 and 181, also conferred significant escape from day 111 plasma (IC_50_ = 1∶69). An infrequent G145E substitution at day 111 had only an intermediate effect on virus neutralization (IC_50_ = 1∶523) and did not expand to greater prevalence at later dates. Surprisingly, amino acid substitutions at the amino- and carboxy-terminal base of V3 (N300H, R327K/E332K), which did not appear until day 132 in the SGA sequences and did not predominate until day 181, also led to complete neutralization escape from day 111 plasma (IC_50_ = 1:<20–1∶24). Other V3 base mutations, including T295N, and R327K and E322K individually, conferred partial escape (IC_50_ = 1∶332–1∶532). These findings raised the possibility that the base of V3 could comprise a discontinuous epitope recognized by the initial Nab response with V1 contributing directly or indirectly to Nab recognition and escape.

Neutralization patterns of day 181 plasma revealed continued evolution of fine Nab specificities (Table 1). Day 181 plasma potently neutralized both the T/F virus (IC_50_ = 1∶2432) and the Env clones containing V1 mutations (E146K, IC_50_ = 1∶3288; E146G, IC_50_ = 1∶1718) that conferred resistance to day 111 plasma Nabs. In contrast, mutations located in the V3 base (N300H, R327K/E332K), with or without the V1 mutations, conferred escape from day 181 plasma. Subsequent mutations in the V1 sequence motif at day 412 (N139T/E146T/M147T), however, also led to escape from earlier plasma antibodies including those in day 111 and day 181 plasmas (IC_50_ = 1∶37, 1∶186, respectively). These findings are consistent with a well-described cycle of Nab development and rapid virus escape [6], [30], [31], [33], [42], [43].

#### CH77

In contrast to the robust autologous Nab titers directed against CH40's T/F Env, CH77's plasma Nab neutralized its autologous T/F virus with a relatively low titer of 1∶38 at day 102 and only modestly higher at day 159 (1∶100) (Figure S1). Despite the lower magnitude of titers, plasma antibody selected for complete replacement of the T/F virus in the putative V2 Nab epitope at the day 102 time point (Figure 3; Table 1). The lower antibody titers made it more challenging to detect differences between Nab sensitivity (highest titers of 1∶38) and Nab resistance (lowest level of detection 1∶20 in our assay system). Nevertheless, the V2 region at day 102 displayed nine different polymorphisms over a five amino acid span; eight of these polymorphisms were tested for effects on virus neutralization sensitivity and all conferred statistically significant escape from day 102 plasma Nab, with titers less than 1∶20 in each case (Figure 3B, Table 1). By day 159, plasma Nab titers against the T/F virus had risen to 1∶100, and the V2 mutations continued to confer some degree of escape. This was corroborated by analyses performed with 6 month consensus viruses with and without putative Nab resistance mutations T187aN (in V2) and D230E (in C2) (Figure 1). As with CH40 viruses, CH77 viruses employed multiple pathways to escape Nab pressure at the V2 site, and the mutations that came to dominate the quasispecies at each time point were the most neutralization resistant (Table 1). The V1 mutation, which never completely replaced the T/F virus through the first 159 days, conferred resistance to day 102 plasma but little or no resistance to day 159 plasma. The C2 mutations, which accrued later than the V2 mutations and fully replaced the T/F sequence by day 159, conferred modest escape from both day 102 and 159 plasma Nabs.

#### CH58

Subject CH58 also developed a relatively delayed and low titer autologous Nab response; the first detectable plasma neutralization occurred at day 154 post-seroconversion with titers of 1∶48 (Figure S1 and Table 1). Again, despite the low titers of Nabs, there was virtually complete replacement of the T/F virus by variants with changes in C2, C3 and/or V4. Site-directed mutants designed to distinguish the effects of the predominant mutations present at days 154 and 350 were tested, revealing that the C2, C3 and V4 mutations all conferred significant escape from day 154 plasma Nab (Table 1). By 350 days, autologous Nab titers against the CH58 T/F virus rose to 1∶243 and contemporaneous C3 and V4 mutations showed clear evidence of escape. In addition, there appeared to be linkage between the mutations in C3 and V4 at day 154, with a trend toward sequences having either a C3 mutation or the G410E substitution in V4. This could be demonstrated in the full-length *env* gp160 SGA sequences and inferred from the partial *env* SGA sequences. These results suggest that early CH58 Nabs, despite their low titers of 1∶48 or less, recognized a conformational epitope involving C2, C3 and V4 and selected for virtually complete virus escape by day 154.

### Autologous HIV-1 monoclonal antibodies recapitulate the early polyclonal plasma Nab response in subject CH40

To further define the epitope specificities of the early Nab responses in subject CH40, mAbs AbCH83 and AbCH84 were generated from day 132 B cell cultures by screening for neutralization of CH40 T/F virus. Both mAbs utilized VH 3–30 and Vκ 3–15 VH and VL families, respectively, and were clonally related (Tsao, CY et al. manuscript in preparation). Both AbCH83 and AbCH84 bound well to CH40 T/F Env gp140 (EC_50_ = 0.2 and 0.07 ug/ml, respectively) and both neutralized the CH40 T/F Env pseudovirus potently (IC_50_ = 0.075 and 0.034 ug/ml) (Table 1). AbCH83 and AbCH84 were strictly strain-specific, failing to neutralize heterologous viruses including CH77 and CH58 at concentrations as high as 10 ug/ml (not shown). When tested against the panel of CH40 site-directed mutants shown in Table 1, AbCH83 and AbCH84 demonstrated patterns of neutralization that were strikingly similar to each other and to the day 111 CH40 plasma (Table 1). For example, AbCH83 and AbCH84, like the day 111 plasma, potently neutralized the T/F virus, and V1 and V3 mutations that conferred resistance to day 111 plasma (E146K, E146G, N139T/E146T/M147L, R327K/E332K, and N300H) also conferred resistance to the two mAbs (Table 1). Mutations representing minor sequence variants that conferred partial escape from day 111 plasma also conferred similar degrees of partial escape from AbCH83 and AbCH84 (G145E, N144K, T295N). A site-directed K160N mutant of the CH40 T/F virus did not alter neutralization sensitivity to plasma Nabs or to the two mAbs (Table 1). Interestingly, the C-terminal V3 mutations R327K and E332K together conferred complete escape to each mAb and to day 111 plasma antibodies, but neither mutation alone conferred resistance to the two mAbs and only partial resistance to polyclonal antibodies in plasma. Thus, the two mAbs AbCH83 and AbCH84 nearly recapitulated the polyclonal Nab reactivity in plasma at the day 111 time point, suggesting that the latter was monospecific and directed to a single epitope distinct from that recognized by PG9/PG16/2909 [44].

### Protein adsorptions show that early monotypic plasma Nab responses target conformational epitopes

**Figure 5:**
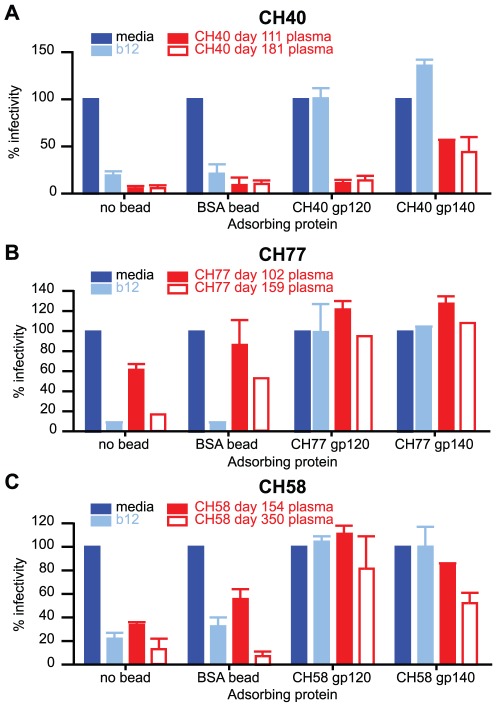
Adsorption of plasma Nabs by autologous T/F Env monomers and trimers. Plasma from CH40 (A), CH77 (B), and CH58 (C) was incubated with magnetic-bead bound gp120 or tethered gp140 protein corresponding to the T/F sequence from each subject. Beads were removed and neutralization assessed by TZM-bl assay (BSA, bovine serum albumen; b12 broadly neutralizing mAb positive control). Results are the mean +/− SD of three independently performed experiments each performed in duplicate.

To distinguish Nab reactivity targeting linear versus conformational (discontinuous) epitopes, we performed competition and adsorption assays with linear Env peptides, full-length gp120 proteins, and full-length tethered gp140 proteins corresponding to the sequences of the autologous T/F viruses, and as controls, heterologous peptides and proteins and randomly shuffled peptide sequences. First, we used overlapping linear peptides spanning complete variable loop and constant region sequences where Nab escape mutations first arose (V1 in CH40; V1 and V2 in CH77; and C2 and V4 in CH58). At concentrations of 25 ug/ml, none of the linear peptides reduced the neutralizing activity in patient plasma (data not shown). As a positive control, we showed that 25 ug/ml of HIV-1 V3 peptides could inhibit V3-targeted Nabs in the same TZM-bl assay [45]. To determine if Nabs in patient plasma recognized epitopes presented on gp120, we performed adsorption assays using autologous gp120 Env monomers and tethered gp140 Env trimers. These Env proteins were attached to magnetic beads, incubated with patient plasma, and then removed before performing neutralization assay because Env alone can neutralize HIV-1 infectivity by binding cell surface CD4. The mAb b12, previously shown to neutralize all three subjects' T/F Envs (IC_50_ = 0.7–1.5 ug/ml), served as a positive control to assess the conformational and antigenic integrity of the synthesized gp120 and gp140 glycoproteins. As shown in Figure 5, b12 at a concentration of 10 ug/ml reduced viral infectivity to below 25% of control in each subject. This inhibitory effect was completely eliminated by preadsorption of b12 with either the Env monomer or Env trimer from each subject, indicating that the gp120 and gp140 proteins had intact b12 binding sites. For CH40, the gp120 Env monomer was ineffective at adsorbing plasma Nab. The tethered gp140 Env trimer, however, adsorbed neutralizing activity allowing infectivity to rise to 57% against day 111 plasma and 45% against day 181 plasma (Figure 5A). These data suggested that CH40's earliest plasma Nab recognized a conformational epitope that is best displayed on trimeric Env, either on contiguous components of neighboring monomers or within a single Env monomer that is dependent on trimeric Env for appropriate presentation.

For CH77 and CH58, weaker autologous Nab titers made the effects of adsorption more difficult to discern, but in both cases, the Env monomers and Env trimers were equally effective at adsorbing neutralizing activity (Figure 5B,C). For CH77, the baseline infectivity was high for the low titer day 102 plasma (>60%), and increased with non-specific binding to the BSA-coated bead, but both the Env monomer and trimer further increased this to >100%. CH77's day 159 plasma demonstrated more clearly that the Env monomer and trimers could both effectively bind and adsorb plasma Nab, with greater differences between negative controls and the Env-coated beads. The adsorption experiment in CH58 demonstrated similar findings; both the gp120 monomer and the gp140 trimer adsorbed neutralizing activity equally (Figure 5C). The ability of gp120/gp140 proteins, but not linear peptides, to adsorb neutralizing activity suggests that the earliest plasma Nabs from CH77 and CH58 targeted conformational epitopes that did not require quaternary structure for effective presentation.

### Structural modeling of Nab epitopes

**Figure 6:**
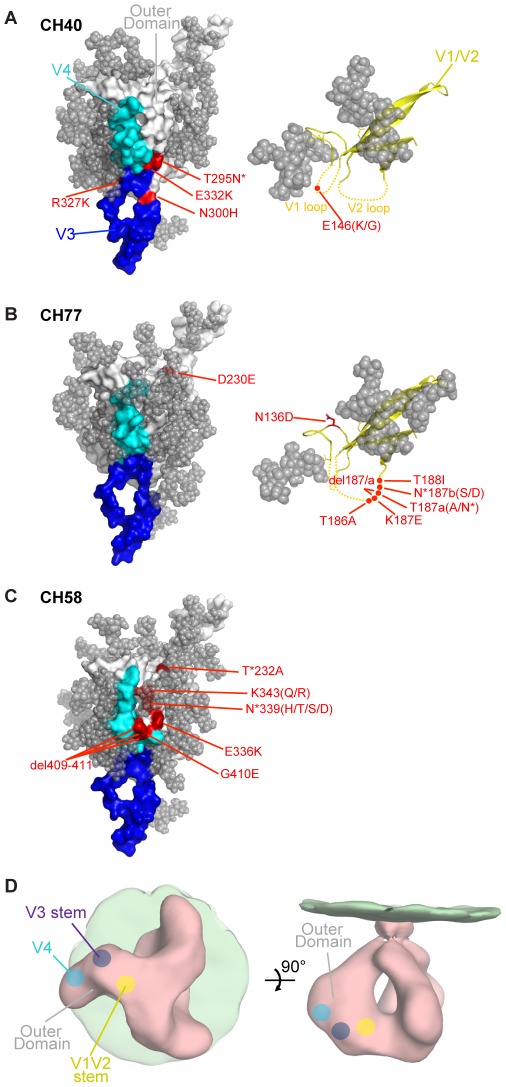
Env models. Models of gp120 molecules from (A) CH40, (B) CH77, and (C) CH58 are shown as white surface projections, with the V3 and V4 loop regions colored in blue and cyan. Models of the V1/V2 regions of CH40 and CH77 are shown as yellow ribbons with parts of the variable V1 and V2 loops shown as dotted lines. Potential N-linked glycans are modeled as grey spheres. Nab escape mutations are shown in red (HXB2 numbering), with mutations removing or adding a potential N-linked glycosylation site marked with an asterisk. (D) Schematic of putative V1/V2 (yellow dot), V3 (blue dot), and V4 (cyan dot) locations on the Env trimer.

Escape mutations were analyzed in the context of a relatively complete model of gp120 that was assembled from crystal structures of core gp120 with V3 and core gp120 with N and C termini (Figure 6A–C, left panels) [46]–[48]. This model lacked only the V1/V2 region, and escape mutations in V1/V2 were thus modeled with the scaffolded structure of V1/V2 [49] (Figure 6, right panels). Despite the availability of atomic-level structures of each of these component portions of gp120, the overall conformation of gp120 in the context of the functional viral spike is still unknown, and so the positions of escape mutations in the spike were inferred from lower resolution electron microscopy results (Figure 6D) [50]–[52]. For CH40, Nab escape mutations were observed at the amino- and carboxy-termini of the V3 region, and included an additional PNLG site at residue 295; escape mutations in the V1 loop were also observed. Phenotypically-proven escape mutations in V1 predominated at the earliest time point (day 111) (Figure 2; Table 1) and evolved through 412 days of follow-up, indicating that these mutations made a major contribution to neutralization escape. A synergistic effect between V1/V2 and V3 neutralization escape mutations has been noted in other contexts [32]–[34], [41], and these two epitopes are spatially close on the low resolution viral spike (Figure 6D). The ability of trimeric gp140, but not monomeric gp120, to absorb neutralizing activity from the CH40 sera (Figure 5A), is consistent with this interpretation and implicates a conformational epitope involving the V1 and V3 regions that requires quaternary protomer interactions for its integrity.

For CH77, the escape mutations appeared predominantly in V2, with a number of different V2 mutations selected, several of which alter a PNLG site. Escape mutations were also selected in the V1 and C2 regions and these involved glycan modifications as well. On the trimer structure (Figure 6D), the C2 and V1/V2 regions are not spatially close, suggesting that that the mutations may have had conformational influence on distant sites or that they were selected to escape different antibody responses. For CH58, escape mutations appeared primarily on the gp120 outer domain in the C2, C3 and V4 regions. These mutations map to a glycosylated outer vertex of the viral spike, which is relatively restricted in space, and therefore likely represents a single epitope not expected to be quaternary in nature. This interpretation is supported by the finding that monomeric gp120 absorbs neutralizing activity from this serum (Figure 5C).

### Deep sequencing of phenotypically confirmed Nab epitope regions

SGA-based sequencing provides a proportional representation of plasma viral populations but with limited sensitivity due to practical constraints of gene-wide sequencing [10]. With a sample size of 30 sequences, there is a 95% probability of detecting a variant that comprises ≥10% of the population [10]. To detect variants comprising substantially less than 10% of the circulating plasma virus, we used parallel allele-specific sequencing (PASS) and 454 pyrosequencing. PASS involves PCR amplification within a polyacrylamide gel using modified primers and fluorophore-labeled nucleotides to distinguish single nucleotide polymorphisms in each amplicon [53]. Using PASS, we characterized hundreds to thousands of sequences per time point over a six nucleotide span in CH40 and CH77 corresponding to known Nab escape mutations. Due to the low plasma viral load of CH58, PASS analysis was not feasible in this subject. For CH40, SGA sampling (14 sequences) of day 45 plasma revealed only T/F sequences in V1, whereas PASS detected 1.1% (5/492) of sequences with the E146K Nab escape mutation and 0.4% (2/492) of sequences with an M147L mutation (Figure 2B; Table S1). PASS yielded similar increases in sensitivity in detecting Nab escape mutations in subject CH77, where SGA sequencing revealed 100% T/F virus at day 32 in V2 and PASS identified a small variant population (0.2%) of the predominant (T187aN) Nab escape variant seen in the subsequent time points (Figure 3B; Table S2).

**Figure 7:**
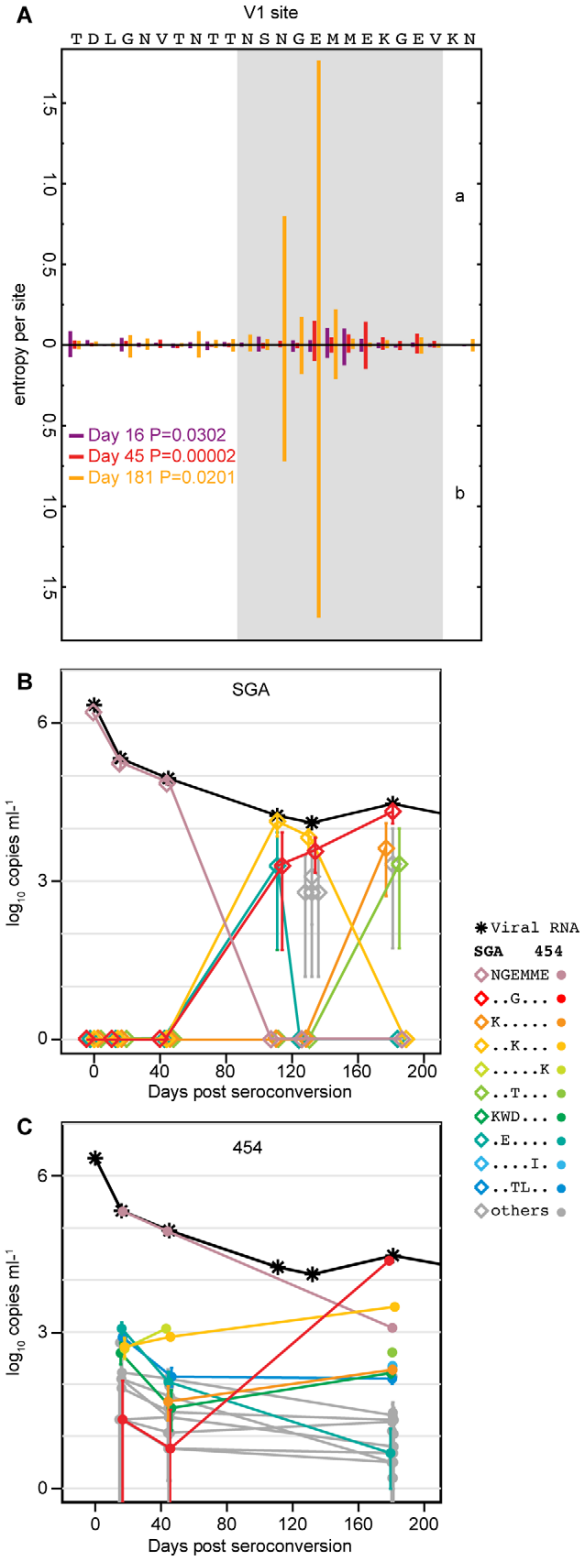
Sequence entropy and viral dynamics at the CH40 V1 Nab epitope. (A) 454 sequence entropies inside (grey) and outside (white) of a 12-amino acid Nab epitope region in V1. Bar length indicates Shannon entropy computed bidirectionally (a and b) (one-sided Wilcoxon analysis). (B and C) Plasma viral RNA (black asterisks) consist of epitope variants in various abundances as determined by SGA or 454 sequencing. Error bars depict 95% confidence intervals from the binomial distribution.

454 pyrosequencing extended these analyses in subject CH40 where the immunodominant V1 epitope was analyzed over time. Sequences from days 16, 45 and 181 post-seroconversion were amplified and bi-directional reads from two amplicons spanning the V1 loop of the T/F sequence were codon aligned and analyzed. The number of high-quality, interpretable reads spanning V1 ranged from 10,275 to 22,344 (Table 2). These V1 sequences yielded 81, 110 and 249 unique nucleic acid sequences and 63, 77 and 246 unique amino acid translations, for days 16, 45 and 181, respectively (Table 2). Compared to SGA and PASS sequences, 454 sequencing substantially increased the sensitivity for detection of rare variants (Figure 7). Over a six amino acid span covering the V1 Nab epitope region NGEMME (HXB2 positions 144–149), SGA detected only the T/F among fourteen day 45 plasma viral genomes (Table S1) and PASS detected two variant sequences in 7 of 459 viral genomes (Table S1), whereas 454 pyrosequencing detected 18 variants among 481 sequences that differed at day 45 from the T/F genome.

**Table 2:** 454 pyrosequencing sequence characteristics.

	Reads^a^	Clean^b^	NTs^c^	Trans^d^	AAs^e^	Reads Excluded	%Reads Excluded
day 16	10,275	10,215	81	10,215	63	60	0.58
day 45	15,487	15,420	110	15,420	77	67	0.43
day 181	22,344	21,255	249	21,252	246	1089	4.87

a Total number of reads that spanned V1.

b Number of reads used for analysis excluding reads with multiple deletions and inversions from sequencing error.

c Number of distinct nucleotide sequences among the cleaned reads.

d Number of reads whose amino acid translations exclude incomplete and stop codons.

e Number of distinct amino acid sequences among the cleaned reads, excluding reads with incomplete and stop codons.

To test whether the detection of rare Nab escape variants by 454 sequencing constituted statistically significant evidence of selection at this epitope, we compared this region of *env* with an adjacent control region. The Nab epitope region (NSNGEMMEKGEV) corresponded to Env codons 142–154 (Figure 7B) and contained the phenotypically-confirmed early Nab escape mutations (Figure 3 and Table 1). We compared this region with an adjacent control region composed of the remaining 12 amino acids falling between the two conserved cysteine residues that bound the V1 loop. These two regions had similar sizes, were both located in a variable region, and were covered within the same 454 reads, thus precluding issues of differential sequence coverage and error rates. We compared the two regions using three different statistical methods. *Entropy comparisons:* Using Shannon entropy, a simple measure of variation in DNA and protein sequence alignments that reflects both the number of variants and their distribution, we computed the amino acid entropies per site and then compared the entropies inside and outside of the Nab epitope region. Entropies increased with time (Figure 7B) and were significantly greater inside than outside of the Nab epitope region for each time point, including just day 16 post-seroconversion (p = 0.0302) and day 45 (p = 1.97×10^−5^ by one-sided Wilcoxon rank-sum test). This significant difference in entropies resulted from a rank-based statistic, which is insensitive to the extremes of values, as seen as large peaks at N143 and E145 (Figure 7B). This indicates greater variability among nearly all sites inside compared to outside the Nab epitope region. *Positive Selection:* To detect evidence of positive selection pressure, we assessed the ratio of non-synonymous to synonymous substitution rates (dN and dS, respectively). When dN<dS, negative selection is evident; conversely, dN>dS indicates positive selection. The large sample sizes that result from 454 pyrosequencing are computationally intractable for most established procedures that test for positive selection. We used SNAP (Synonymous Non-synonymous Alignment Program), which corrects for alternative mutational pathways in a codon and performs efficiently for large sequence sets. Again, we compared the 12 amino acid Nab epitope region with the remaining 12 amino acids in V1 and computed the distance-corrected synonymous and non-synonymous substitution rates with SNAP. For each amplicon sampled, we summarized SNAP results as contingency tables, wherein columns indicate dN<dS or dN>dS and rows indicate sites inside or outside the epitope. We then populated the table with counts for each sequence relative to the T/F sequence (excluding cases where dS = dN) and used one-sided Fischer's exact tests to evaluate whether the epitope was enriched for non-synonymous substitutions (Table 3). These comparisons indicate significant increases in positive selection within the epitope at day 45 (p = 0.013) and day 181 (p = 0.002), with a trend at day 16. *Poisson Model:* Following the method of Giorgi et al. [54], we used a simple model of sequence evolution to test for homogeneous infection. The model gives a null hypothesis of Poisson-distributed intersequence distances. For the single variant transmission of CH40, rejecting the null model indicates that selection is present in the sequences sampled. Because APOBEC hypermutations violate model assumptions, the test is repeated without APOBEC mutated sites. With APOBEC mutations excluded, the Poisson model test results indicate simple evolution and no selection in reads sampled at day 16 and day 45 (P>0.9, Table 4). When APOBEC sites are retained in the analysis, the Nab epitope fails to conform to the Poisson (P<10^−9^, Table 4), consistent with selection facilitated by APOBEC. By 181 days post-screening, the Poisson model is rejected regardless of whether or not APOBEC hypermutations are excluded (P<10^−9^, Table 4). All three methods indicated that variation within the putative V1 Nab epitope region was statistically significantly enriched over background mutations; selection for Nab escape mutations was unequivocal by all methods of analysis at day 45 and day 181 and supported at day 16 by an increase in entropy within the epitope region (p = 0.03, Figure 7).

**Table 3:** Comparison of synonymous and non-synonymous substitutions inside and outside of the V1 Nab epitope region.

				Inside Nab Epitope		Outside Nab Epitope	
Sample	P^a^	OR^b^	CI^c^	dN<dS^d^	dN>dS^e^	dN<dS^f^	dN>dS^g^
day 16	0.250	0.609	<1.665	8	37	10	28
day 45	0.013	0.372	<0.079	15	60	19	28
day 181	0.002	0.457	<0.733	35	197	37	95

a P-value from one-sided Fisher's exact test.

b Odds ratio.

c 95% confidence interval.

d,e Number of nucleotide sequence variants with mutated sites inside the V1 epitope region, where synonymous substitution rate exceeds non-synonymous substitution rate, and non-synonymous substitution rate exceeds synonymous substitution rate, respectively.

f,g Number of nucleotide sequence variants with mutated sites in V1 outside the epitope region, where synonymous substitution rate exceeds non-synonymous substitution rate, and non-synonymous substitution rate exceeds synonymous substitution rate, respectively.

**Table 4:** Comparison with Poisson model of random sequence evolution.

Sample	Reads^a^	APOBEC^b^ positions	NTs^c^	meanHD^d^	Max HD^e^	P^f^
**day 16 V1**	10,215	included	73	0.122	4	0.006
		excluded	55	0.063	4	0.988
**day 16 Nab epitope** g		included	37	0.082	4	<10−9
		excluded	23	0.030	2	0.494
**day 16 non-epitope** h		included	37	0.040	3	0.880
		excluded	31	0.033	3	0.978
**day 45 V1**	15,420	included	73	0.125	6	<10−9
		excluded	55	0.043	4	0.966
**day 45 Nab epitope**		included	37	0.100	6	0
		excluded	23	0.023	4	0.525
**day 45 non-epitope**		included	37	0.025	2	0.530
		excluded	31	0.018	2	0.711
**day 181 V1**	22,304	included	73	1.170	10	<10−9
		excluded	54	0.367	7	<10−9

a Number of reads in Poisson distance distribution.

b APOBEC sites were included or excluded from the analysis.

c Number of nucleotides used to compute pairwise sequence distances.

d Mean Hamming distance in pairwise sequence distance distributions.

e Maximum Hamming distance in pairwise sequence distance distributions.

f P-value for goodness-of-fit to the Poisson distribution by chi-squared test; where P>0.01, the sample fits the Poisson null model.

g Nab epitope includes the 12 amino acids encompassing phenotypically-proven Nab escape mutations; see Figure 7.

h Non-epitope includes the 12 remaining amino acids in V1 not included in Nab epitope region; see Figure 7.

## Discussion

We used three increasingly sensitive DNA sequencing methods – SGA, PASS and 454 – to look for genetic evidence of Nab selection on the evolving HIV-1 quasispecies. By three to six months post-seroconversion, SGA sequencing identified a set of candidate Nab escape mutations, which in every subject was discontinuous and could be distinguished from CTL escape mutations [7]. Each of the candidate Nab escape mutations that we inferred from SGA sequencing was shown phenotypically to confer significant (2 to >70 fold) resistance to early Nabs (Table 1). Remarkably, at the time of initial detection of Nab titers, regardless of titer, the virus quasispecies in each subject demonstrated complete or near complete replacement of the T/F sequence by escape mutants at their respective Nab epitopes. This indicated a pre-existent Nab response. PASS analysis corroborated this finding by revealing genetic evidence of Nab escape significantly earlier at just 45 days and 32 days post-antibody seroconversion in subjects CH40 and CH77, respectively (Tables S1 and S2). This was at a time point when Nab titers to each T/F virus were undetectable at a 1∶20 plasma dilution in the TZM assay (Table 1). Nabs at this early time point were also below the level of phenotypic detection when tested in the sensitive A3R5 cell-based virus entry assay [55] (D. C. M., unpublished). In subject CH40, where viral loads were highest and deeper sequencing could be done, 454 analysis identified a much larger number of variants in the V1 epitope region of the T/F virus sequence at a still earlier time point 16 days post-seroconversion as well as at 45 and 181 days post-seroconversion (Table 2). The 454 data further suggested a role for APOBEC mutations facilitating this escape, since at days 16 and 45 post-seroconversion the V1 Nab epitope region was enriched for mutations at APOBEC motifs (Table 4). APOBEC and Vif function have been implicated in virus escape from early CTL immune pressure [56], and our results suggest that APOBEC may play an analogous role in the dynamics of early Nab escape at certain epitopes. It is possible that the increased genetic diversity in V1 arising from APOBEC mediated polymorphisms facilitated more rapid escape in this region than in other regions of the Nab epitope. Overall, a combination of SGA, PASS and 454 pyrosequencing enabled the genetic detection of Nab escape variants significantly before Nabs rose to titers detectable in the TZM assay. This enhancement in detection amounted to 95 days in subject CH40, 70 days in subject CH77, and 109 days in subject CH58. Future studies in other subjects with more narrowly spaced sampling intervals may better define these windows.

In each subject we found an early monospecific Nab response directed toward a single conformational epitope that was unique to each T/F virus strain. This was demonstrated most clearly in subject CH40 where polyclonal plasma antibodies and autologous mAbs targeted essentially the same epitope at the base of V3 (depicted in Figure 6). Previous studies have reported epitopes in this region of gp120 to be immunogenic and a target of both broadly and narrowly reactive neutralizing mAbs [37], [57]. Interestingly, we observed that the binding of both AbCH83 and AbCH84 to autologous CH40 Env gp140 as assessed by Biocore analysis could be blocked by the potent and broadly neutralizing PGT 121 mAb, which in other contexts is dependent on N332 [57] (B.F.H., unpublished). The early Nab response in CH40, unlike responses in CH77 and CH58, targeted an epitope dependent on trimeric Env for structural integrity. Thus, structural modeling and empirical analyses suggested that in subject CH40, virus escaped Nab pressure indirectly by early mutations in V1 and directly through mutations in the putative V3 epitope, indicating a close association between V1 and V3 in the context of the native functional Env trimer. In all three subjects, we identified Nab epitopes involving unique sites on the Env glycoprotein, with continuous virus evolution at the respective epitopes, without evidence of broadening of the Nab response to additional sites on the Env trimer over the first year of infection (Figures 2–4). In CH77, escape occurred predominantly in V2, where the addition of PNLG site conferred Nab escape at a likely protein epitope. In CH58, modeling suggested that early Nabs targeted a single conformational epitope involving the Env outer domain, with escape arising through the loss of any of several component glycans. Thus, in each subject, virus employed glycan shifts as well as gain or loss of glycans to mediate escape from the sequential rounds of the Nab response. These findings, in conjunction with reports of monospecific early Nab responses in subtype C infection [58], [59], suggest that individual immunodominant regions of Env, specific to the unique conformation of each T/F Env, are targeted by early Nab responses.

The observation that very low level Nab titers can impede virus entry and select for virus escape *in vivo* is consistent with recent findings of selection for SIVmac251 and SIVsmE660 Nab escape mutations in early-chronic infection of rhesus macaques by low titers of Nabs [60], the association of low-titer Nabs with protection against SIVsmE660 challenge in the nonhuman primate (NHP) model [26], and results from low dose mucosal NHP challenge models in which concentrations of Nabs corresponding to modest *in vitro* titers were able to effectively prevent SIV acquisition [24], [25]. To our knowledge, however, this is the first demonstration in human HIV-1 infection that very low titers of Nabs in the range of 1∶50 to 1∶20 or even lower can impede virus replication and select for virus escape. To explore quantitatively the *in vivo* activity of early Nab responses, we employed a mathematical model to estimate the proportion of *de novo* infection events blocked by Nabs, or the Nab efficacy (see Figures S3, S4 and Dataset S1). The results, which represent minimum estimates, ranged from a low of 19.6% to a high of 35.2% and represent a Nab response that is sufficiently potent to drive replacement of the T/F virus within several weeks (Figure S4 and unpublished data). Our conservative modeling likely underestimated true Nab efficacy because we utilized minimum estimates for biological parameters with uncertain quantities and did not account for potential fluctuations in Nab efficacy. Future studies where sampling time points are better structured for evaluating dynamic changes in Nab titers and viral quasispecies composition would allow for greater precision in estimations of Nab efficacy *in vivo* and a better understanding of the kinetics of Nab development.

These caveats notwithstanding, the data raise the possibility that in the setting of sexual transmission, where the risk of infection per coital act is low and the number of transmitted viruses responsible for productive clinical infection is typically one, a vaccine that elicited Nabs of sufficient breadth but at titers as low as 1∶50 to 1∶20 or possibly even lower could have a demonstrable protective effect. It is possible that such a low titer neutralizing activity in vaccinees from the Thai RV144 trial could have contributed to the observed 31% protective effect of the vaccine [27], [61], [62].

The rates of Nab-driven T/F sequence replacement are more rapid than previously reported [6], [30], [32], [33], [35], [36], [40], [42], [59] but substantially slower than rates of loss due to the initial CTL responses [4], [63] A unique aspect of the present study is that we could directly compare the rate of Nab escape with the rate of CTL escape in the same three subjects [7]. Based on SGA analyses, we previously observed virtually complete replacement of the T/F virus population at defined CTL epitopes within 45 days (CH40), 14 days (CH77) and 45 days (CH58) of antibody seroconversion [7], [8]. This contrasts with 111, 102 and 154 day intervals shown in the present report for Nab escape. Similarly, in a 454 pyrosequencing analysis of CTL escape kinetics, we previously observed in subject CH40 a 1% replacement of T/F sequences just prior to antibody seroconversion (corresponding to day 0 in the present study), a 52% replacement by day 16, and a 99.4% replacement by day 45 [38]. Comparable numbers for Nab escape variant frequencies in subject CH40 in the present study were <1%, 2% and 3%, respectively, again highlighting the much faster rate of CTL escape compared with Nab escape. Furthermore, in the former study, we found that the average rate of HIV escape from CTL responses in acute infection to be 0.17 day^−1^ with a maximum of 0.42 day^−1^ [7], [38]. The average rate drops to 0.03 day^−1^ by 100 days post seroconversion [64]. This slower rate of virus escape from chronic CTL responses is similar to that of contemporaneous Nab responses measured in the current study, suggesting that Nabs could contribute along with CTLs to virus containment during this later time period. In acute infection of unvaccinated subjects, however, Nab responses likely contribute negligibly to early virus containment.

The costs to replication fitness associated with virus escape from autologous Nab responses have not been well characterized but could contribute to partial virus containment at setpoint viremia. Fitness costs of Nab escape mutations have frequently been considered to be minimal [35], [65], but Derdeyn and colleagues described a Nab escape mutation in V2, which when placed in the autologous T/F virus backbone, conferred a measurable fitness cost [58]. Morris and colleagues [31] similarly noted transient decrements in plasma virus load coincident with the development of strain-specific Nabs. We studied Nab escape mutations within the context of a 6 month consensus IMC so the effects of compensatory mutations could be accounted for and so mutations resulting from escape from Nabs could be distinguished from those resulting from escape from CTLs. Our analyses suggested that Nab escape mutations conferred reductions to replication fitness ranging from 0 to 24%. This corresponds to an estimated average impairment to virus entry due to early strain-specific Nabs of as much as 31.3% to 48.8%.

Finally, we note that the exquisite sensitivity and rapid adaption of HIV-1 Env to Nabs contrasts with recent observations for the HIV-2 Env, where high-titer Nabs register little effect on *env* evolution or Env Nab escape [66]–[68]. A biological explanation for these differences is not obvious. For HIV-1, the enhanced sensitivity and rapid adaptation to Nab pressure *in vivo* provides an explanation for the HIV-1 Env's propensity to maintain a fully assembled glycan/conformational shield [6]. Paradoxically, it is this enhanced sensitivity of HIV-1 to Nabs *in vivo* that appears to be responsible for its vaunted ability to resist neutralization by all but the most broadly reactive and potent Nabs. Another provocative implication of the current study is that, *in vivo*, Nabs impede HIV-1 spread whether this is occurring by ‘cell-free’ or ‘cell-to-cell’ mechanisms. This is at odds with the suggestion that ‘cell-to-cell’ spread of HIV-1 provides a mechanism for replicating virus to escape Nab or antiretroviral drug pressure [69], [70]. Further investigation is needed to resolve this question.

## Materials and Methods

### Ethics statement

This study was conducted according to the principles expressed in the Declaration of Helsinki. It was approved by the Institutional Review Boards of the University of Pennsylvania, the University of Alabama at Birmingham, the University of North Carolina and Duke University. All subjects provided written informed consent for the collection of samples and subsequent analysis.

### Experimental strategy overview

The experimental strategy was first to define the kinetics of appearance of autologous and heterologous Nabs in each subject by a conventional single-cycle virus entry assay [6], [71]. Next, we performed an in-depth SGA-based analysis of plasma viral *env* gp160 RNA sequences at serial time points beginning prior to antibody seroconversion and extending beyond the first year of infection. The SGA approach allowed us to look for amino acid selection across intact *env* gp160 genes not accounted for by CTL-driven virus escape [7] that might reflect Nab-mediated virus escape. Next, putative Nab epitopes were corroborated by cloning and analyzing full-length infectious molecular clones (IMCs) corresponding to T/F and consensus 6 month sequences and by performing site-directed mutagenesis on T/F *env* genes so as to introduce individual putative escape mutations arising in the first year of infection for phenotypic testing against sequential patient plasma specimens and monoclonal antibodies. Plasma samples containing neutralizing activity were adsorbed with autologous or heterologous Env peptides or polyproteins to distinguish linear from conformational Nab epitopes. This was followed by deeper sequence analyses using parallel allele-specific sequencing (PASS) and 454 pyrosequencing to identify the earliest genetic signatures of Nab escape at confirmed epitopes. T/F and consensus 6 month IMCs, with and without Nab escape mutations, were evaluated for *in vitro* replication kinetics to access fitness costs of Nab escape, and structural and mathematical models were used to interpret data within the context of viral Env structure and replication kinetics *in vivo*.

### Study subjects

Peripheral blood samples were obtained from subjects 700010040 (CH40), 700010058 (CH58), and 700010077 (CH77) after obtaining informed consent under the Duke University and University of North Carolina human use review boards. All subjects were North American men who had sex with men (MSM) who denied injection drug use all were infected with HIV-1 subtype B, and all were antiretroviral drug naïve throughout the study course. At initial sampling, the three subjects were at peak viremia (Fiebig stage II, plasma vRNA+, and Ab−) just prior to HIV antibody seroconversion [10], [72], [73].

### Viral RNA extraction and cDNA synthesis

Viral RNA from each time point was extracted and reverse transcribed to cDNA as previously described [8]. Approximately 20,000 viral RNA copies were extracted using the BioRobot EZ1 Workstation with EZ1 Virus Mini Kit (version 2.0; QIAGEN), and 5,000 vRNA molecules were reverse transcribed using SuperScript III (Invitrogen) and the primer R2.B3R 5′-ACTACTTGAAGCACTCAAGGCAAGCTTTATTG-3′.

### Single Genome Amplification

SGA was performed as described previously [8], [10]. Briefly, cDNA was serially diluted so as to identify a dilution where PCR positive wells constituted less than 30% of the total number of reactions. At this dilution, most wells contain amplicons derived from a single cDNA molecule. PCR reactions used Platinum Taq High Fidelity polymerase (Invitrogen) and nested primers OFM19 and Vif1 (first-round) and EnvA and EnvN (second round) to generate full-length gp160 *env* sequences. To obtain subgenomic sequences containing putative Nab epitopes in CH58, nested primers CH58C2.OutF, CH58C2.OutR, CH58C2.InF, and CH58C2.InR were used to amplify a 554 nucleotide region spanning V1 through C3, and nested primers CH58.C3V4.5outA, CH58.C3V4.3outA, CH58.C3V4.5InA, and CH58.C3V4.3InA were used to amplify a 377 nucleotide region spanning regions V3 through C4. PCR parameters were as follows: 94°C for 2 min, followed by 35 cycles of 94°C for 15 s, 58°C for 30 s, and 68°C for 4 min, followed by a final extension of 68°C for 10 min. The product of the first-round PCR was used as a template in the second-round PCR reaction under the same conditions, but with a total of 45 cycles. The amplicons were inspected on precast 1% agarose E-gel 96 (Invitrogen Life Technologies). All PCR procedures were carried out under PCR clean room conditions. SGA primer sequences:

OFM19: 5′-GCACTCAAGGCAAGCTTTATTGAGGCTTA-3′

Vif1: 5′-GGGTTTATTACAGGGACAGCAGAG-3′

EnvA: 5′ GGCTTAGGCATCTCCTATGGCAGGAAGAA-3′

EnvN: 5′-CTGCCAATCAGGGAAGTAGCCTTGTGT-3′.

CH58C2.OutF: 5′-CCATGTGTACAATTAACCCCACTCTGTGTC-3′

CH58C2.OutR: 5′-CTGTTCTCTTAATTTTGTAACTATCTTC-3′

CH58C2.InF: 5′-GTAGCGAGGGAAAGGAAATGAAGAACTG-3′

CH58C2.InR: 5′-GTGTTATTCCATTGTTCTCTACTAAGGTTAC-3′

CH58.C3V4.5outA: 5′-CTGCTGTTAAATGGCAGTCTAGCAGAAAAAGATATAG-3′

CH58.C3V4.3outA: 5′-CTCATATCTCCCCCTGCAGGTCTGAAGGTC-3′

CH58.C3V4.5InA: 5′-GTACAAGACCCAACAACAATACAAGAAAAAGTATAAC-3′

CH58.C3V4.3InA: 5′-CCTTTGATGGGAGGGGCATACATTGCTTTTC-3′

### DNA sequencing

Amplicons were directly sequenced by cycle-sequencing using BigDye Terminator chemistry (Applied Biosystems). Sequencing reaction products were analyzed with an ABI 3730xl genetic analyzer (Applied Biosystems). Both DNA strands were sequenced using overlapping fragments. Individual sequence fragments for each amplicon were assembled and edited using the Sequencher program 4.8 (Gene Codes; Ann Arbor, MI). Chromatograms containing mixed bases (double peaks) were excluded.

### Sequence alignments

All sequences were manually inspected and aligned in MacClade 4.08 to optimize alignments. Consensus sequences were generated for each individual from the earliest sample (pre-antibody seroconversion, Fiebig Stage II) and longitudinal sequences aligned accordingly. All sequences were deposited in GenBank (accession numbers: JQ957568–JQ957796).

### *Env* gene cloning, sequencing, and site directed mutagenesis

Full-length gp160 *env* genes were amplified by nested PCR from acute infection plasma HIV-1 RNA, cloned, and sequenced to confirm their identity with T/F genomes [6], [10]. Site-directed mutations corresponding to naturally-occurring mutations were introduced with QuickChange site-directed mutagenesis kit (Strategene).

### Generation of Infectious Molecular Clones (IMCs) corresponding to T/F and six month consensus genomes with and without putatitive Nab escape mutations

IMCs of T/F genomes were previously described [8], [39]. SGA-derived sequences from 6 months (159–181 days) post-seroconversion were used to determine a consensus sequence. At polymorphic positions, the majority nucleotide was selected. At positions where there was no single nucleotide representing >50% of sequences, the most prevalent nucleotide was selected. Six month IMCs, with and without putative Nab escape mutations, were constructed by chemical synthesis (Blue Heron) and site-directed mutagenesis by methods previously described [8], [39]. All IMCs were sequence confirmed.

### Neutralization assays

Plasma samples were assayed for Nab activity against IMC-derived virions or Env- pseudotyped virions using a single-round JC53BL-13/TZM-bl pseudotype reporter assay [6]), JC53BL-13 cells were plated and cultured overnight. A total of 2,000 infectious units of each pseudotyped virus were combined with fivefold dilutions of heat-inactivated test plasma or serum and incubated for 1 h at 37°C. Non-HIV-infected heat-inactivated human plasma was added as necessary to maintain a constant overall concentration. The virus-Ab mixture was then added to JC53BL-13 cells, and after 2 days, the cells were lysed, and the luciferase activity of each well was measured using a luciferase assay reagent (Promega, Madison, WI) and an ABI Tropix (Applied Biosystems, Foster, CA). Background luminescence was determined in uninfected wells and subtracted from all experimental wells. Cell viability and toxicity were monitored by basal levels of luciferase expression and by visual inspection. Relative infectivity (percentage of control) was calculated by dividing the number of luciferase units at each plasma dilution by the values in wells containing no test plasma. The dilution of test plasma or serum that inhibited 50% of virus infectivity (IC_50_ titer) was determined using a linear regression-least squares fit method. mAbs were tested for neutralizing activity beginning at 10 ug/ml and proceeding with five-fold dilutions, as previously described [6].

### Isolation of monoclonal antibodies

IgG+ memory B cells were isolated from frozen peripheral blood mononuclear cells (PBMCs) from day 111 after enrollment and cultured at near clonal dilution as described [74]. Cells were obtained from CH40 at 132 days post-seroconversion by selecting CD2−, CD14−, CD16−, CD235a−, IgD− and IgG+ cells through two rounds of separation with magnetic beads (Miltenyi Biotec, Auburn, CA). Cells were then resuspended in complete medium containing 2.5 µg/ml CpG ODN2006 (tlrl-2006; InvivoGen, San Diego, CA), 5 µM CHK2 kinase inhibitor (Calbiochem/EMD Chemicals, Gibbstown, NJ) and EBV (200-µl supernatant of B95-8 cells/10^4^ memory B cells). After overnight incubation at 37°C in 5% CO_2_, 21,600 viable cells were seeded in 96-well round-bottom tissue culture plates at a cell density of 3 memory B cells/well in presence of ODN2006, CHK2 kinase inhibitor and irradiated (7,500 cGy) CD40 ligand-expressing L cells (5,000 cells/well). Cells were re-fed at day 7 and harvested at day 14. The two 96 well supernatants that most effectively neutralized CH40 T/F Env pseudotyped virus in the TZM-bl assay, as previously described [74], were selected for further analysis. RNA from positive cultures was extracted (RNeasy minikit; Qiagen), and the genes encoding Ig V(D)J rearrangements were amplified by RT and nested PCR, and the mAbs expressed as recombinant IgG1 antibodies (designated AbCH83 and AbCH84) as previously described [74]. Recombinant monoclonal antibodies AbCH83 and AbCH84 were assessed for neutralization in TZM-bl neutralization assays against autologous and heterologous pseudotyped viruses and for Env binding with enzyme-linked immunosorbent assays with a panel of autologous and heterologous tier 1 and 2 viruses and viral proteins [74].

### HIV-1 peptides and polyproteins for antibody adsorptions

For CH40, a 26-mer peptide corresponding in sequence to the CH40 T/F virus V1 region and a control 26-mer scrambled peptide were synthesized (New England Peptide, Gardner, MA). For CH77 and CH58, 18-mer peptides overlapping by 10 amino acids were synthesized (Sigma-Aldrich; Medical Research Council Human Immunology Unit, WIMM, Oxford, UK) to match the T/F sequences of interest for CH77 and CH58. For CH77, peptides spanning V1: LTPLCVTLNCTDSNGDS (3284), V2: PIDTKTNTSKYRLISCNT (3292), DVVPIDTKTNTSKYRLIS (3291), and C2: IPIHYCAPAGFAILKCKD (1181), AGFAILKCKDKKFNGTGP (1467), KDKKFNGTGPCKKVSTVQ (3297) were used. For CH58, peptides matching the sequence of the C2: AGFAILKCNNKTFNGTGQ (3549), NNKTFNGTGQCTNVSTVQ (3550), and V4: KANGTTGNDTIILPCRIK (3570) were used. For both CH77 and CH58, two control peptides matching regions other than Env were used [IVYIEYRKIVRQRKIDRL (3512), MQSLYILGIVALVVAAIL (3509)]. Autologous gp120 and gp140-tethered Env glycoproteins were generated as described [49], [75]. HIV-1 gp140 trimeric envelope glycoproteins contained a mutated furin cleavage site and a C-terminal fibritin trimerization domain with 8xHisTag. Mammalian codon-optimized genes encoding the wild type and mutant gp120s and gp140s were synthesized and cloned into the mammalian expression vector pHLSec2 (GENEART AG, Regensburg, Germany). For preparation of each envelope glycoprotein, 500 ug of the plasmid DNA was mixed with 1 ml of 293fectin (Invitrogen, Carlsbad, CA) for 20 minutes before the DNA-293fectin complex was added into 850 ml of FreeStyle 293F cells (1.4×10^6^ cells/ml) in a 2 L shaking flask. After transfection, the cells were returned to suspension incubation for 24 hours at 37°C, 8% CO_2_ and 125 rpm. The culture was fed with 50 ml of the enriched medium CellBoost-5 (HyClone, Logan, UT) and sodium butyrate at final concentration of 2 mM (SIGMA, St. Louis, MO). After 5 days of suspension culture post transfection, supernatant was harvested by centrifugation and filtered through 0.22 µm filter. For the gp120 protein preparation, the supernantant was purified through an affinity column of 17b (made by cross-linking 17b antibody with Protein A plus agarose (Pierce, Thermo, Rockford, IL). For the gp140 protein preparation, the supernantnt was concentrated and buffer exchanged through a Tangential Flow Filtration system (Pall, Ann Arbor, MI) against Ni-binding buffer, and purified through a Ni-NTA resin column (QIAGEN, Valencia, CA). The purified proteins were concentrated and dialyzed against PBS and characterized by SDS-PAGE and immune blotting with anti HIV-1 IgG (HIVIG).

### Protein-paramagnetic bead coupling

The gp120 Env monomers and tethered gp140 trimers were coupled to a solid phase tosylactivated magnetic Dynabeads MyOne beads (Invitrogen) as previously described [76]. One mg of protein was coupled to 50 mg (0.5 ml volume) of tosylactivated magnetic beads. Coupling was performed at 37°C in a total volume of 1.25 ml in coupling buffer (0.1 M sodium borate buffer (pH 9.5) w 1 M ammonium sulfate) with gentle rocking over 8 to 12 hours. The Dyna beads and bound protein were separated from the coupling buffer with a magnet and resuspended with 5 ml of blocking buffer (PBS (pH 7.4) with 0.1% (wt/vol) BSA and 0.05% Tween 20). The beads were then resuspended in 0.5 ml of storage buffer (pBS (pH 7.4) supplemented with 0.1% (wt/vol) BSA, 0.05% Tween 20, and 0.02% sodium azide and stored at 4°C. Stocks of beads coupled with BSA, to assess for non-specific binding, were prepared in the same manner, incubating in the blocking buffer (PBS (ph7.4) with 0.1% (wt/vol) BSA and 0.05% Tween 20) for the initial step.

### Antibody competition and adsorption

Competition assays were performed with linear peptides having sequences described above. Neutralization assays were performed with additional peptide and plasma incubation for 30 minutes at 37°C prior the addition of the 2,000 infectious units of each virus for 1 hour at 37°C. The neutralization assay was then completed as described above with a final concentration/well of each peptide of 25 ug/ml. For the polyprotein adsorption studies, the plasmas were incubated with 12 uls of the protein-bead complex (or BSA-bound beads) for 30 minutes. The beads were removed with a magnet and discarded. This process was repeated an additional 2 times, using a total of 36 uls (0.036 mg) of bead slurry. In previous reports, three rounds of bead adsorption resulted in nearly complete removal of Env-specific antibodies from serum/plasma samples [76]. After the final incubation, the plasmas were centrifuged at 7000 rpm for 7 minutes. Neutralization assays were then performed as described above.

### Parallel allele specific sequencing

The PASS assay was performed as previously described [53]. Briefly, 20 µl of a 6% acrylamide gel mix (1 µM acrydite-modified primer (CH40-rev or CH77-rev), cDNA template, 0.3% diallyltartramide, 5% rhinohide, 0.1% APS, 0.1% TEMED and 0.2% BSA) was cast on a bind-saline (Amersham Biosciences, Piscataway, NJ) treated glass slide. In-gel PCR amplification was then performed with a 300 µl PCR solution (1 µM primer (CH40/77-for), 0.1% Tween-20, 0.2% BSA, 1× PCR buffer, 100 µM dNTP mix and 3.3 units of Jumpstart Taq DNA polymerase (Sigma, St. Louis, MO) under a sealed SecureSeal chamber (Grace Bio-Labs, Inc., Bend, OR) in a PTC-200 Thermal Cycler. PCR conditions were as follows: incubation at 94°C for 3 min, 65 cycles of a denaturing step at 94°C for 30 sec., an annealing step at 56°C for 45 sec., and an extension step at 72°C for 1 min; and one cycle of an additional extension step at 72°C for 3 min. After PCR amplification, single-base extension (SBE) was performed using the fluorescently labeled nucleotides dGTP-Cy3 (PerkinElmer, Waltham, MA), dTTP-Alexa-568 (Invitrogen, Carlsbad, CA), dATP-Cy5 (PerkinElmer, Waltham, MA), and dUTP-Cy5.5 (GE Healthcare, Piscataway, NJ). Sequencing primers (CH40-seq or CH77-seq) annealed just upstream of the mutation site as well as for the next five consecutive bases. The gel was scanned with an Axon GenePix 4300A Microarray Scanner (Molecular Devices, Sunnyvale, CA) and analyzed with Progenesis PG200 (Nonlinear Dynamics, Durham, NC) software. Sequenced nucleotides were determined by comparing each polony's normalized intensity in all four channels. PASS PCR primer sequences:

CH40-rev: 5′Acr-TTTCCCTGGTCCCATGGGTATACTTTTTC-3′ or

CH77-rev: 5′Acr-ATTATTGCCGGGTCTCATACATTTG-3′

CH40/77-for: 5′-CCACAGACCCCAACCCACAAGAAG-3′

PASS sequencing primer sequences,

**CH40:** 5′-TACTAATACCACTAATAGTAACGGG-3′ (nt 6635–6659), 5′-TACTAATACCACTAATAGTAACGGGG-3′, 5′-TACTAATACCACTAATAGTAACGGGA-3′, 5′-TACTAATACCACTAATAGTAACGGGGAA-3′, 5′-TACTAATACCACTAATAGTAACGGGACA-3′, 5′-TACTAATACCACTAATAGTAACGGGAAA-3′, 5′-TACTAATACCACTAATAGTAACGGGGGA-3′

**CH77:** 5′-TTATAAACTTGATGTAGTACCAATAGATACA-3′ (nt 6752–6782)

5′-TTATAAACTTGATGTAGTACCAATAGATACAA-3′

5′-TTATAAACTTGATGTAGTACCAATAGATACAG-3′

5′-TTATAAACTTGATGTAGTACCAATAGATACAC-3′

5′-TTATAAACTTGATGTAGTACCAATAGATACAAA-3′

5′-TTATAAACTTGATGTAGTACCAATAGATACAGA-3′

5′-TTATAAACTTGATGTAGTACCAATAGATACACA-3′

5′-TTATAAACTTGATGTAGTACCAATAGATACAAC-3′

5′-TTATAAACTTGATGTAGTACCAATAGATACAAC-3′

5′-TTATAAACTTGATGTAGTACCAATAGATACAAAA-3′

5′-TTATAAACTTGATGTAGTACCAATAGATACAGAA-3′

5′-TTATAAACTTGATGTAGTACCAATAGATACACAA-3′

5′-TTATAAACTTGATGTAGTACCAATAGATACAACA-3′

5′-TTATAAACTTGATGTAGTACCAATAGATACAAAAA-3′

5′-TTATAAACTTGATGTAGTACCAATAGATACAAAAG-3′

5′-TTATAAACTTGATGTAGTACCAATAGATACAGAAA-3′

### 454 pyrosequencing

RNA was extracted from pelleted virions containing at least 200,000 viral RNA copies using EZ-1 viral RNA kit (Qiagen) from CH40 plasma from days 16, 45, 181, and 412 post-seroconversion. cDNA was synthesized using superscript III reverse transcriptase (Invitrogen) in 5 replicates with the antisense primer 1.R3.B3R. The cDNA was immediately subjected to nested PCR amplification using Platinum Taq DNA Polymerase High Fidelity (Invitrogen). For each time point, 96 replicate PCR reactions (40 µl each) were performed with 5 µl cDNA, using forward primer BKB3F2 and the same reverse primer used for cDNA synthesis (1.R3.B3R). All 96 first round reactions for each time point were pooled and used as template for three inner PCR reactions. Each inner PCR reaction was performed with 32 replicates (40 µl each) using 5 µl of pooled first round template, and specific primers that incorporated a 4 base identification tag as well as a 19 base 454 adaptor sequence. Agarose gel-run PCR amplicons were visualized with crystal violet/white light and then subjected to 454 sequencing. Each sample was run on a separate picotiter plate with GS-FLX titanium reagents. All amplicons were agar-gel purified, eluted in EB buffer (QIAquick gel extraction kit), and visualized with gentian violet. Directional reads were codon-aligned from two amplicons that span the V1 loop to the T/F sequence and reviewed for sequencing errors as described previously [38]. The forward and reverse reads had similar variant frequencies and entropies per site (P>0.8 by Wilcoxon test) and thus were pooled to increase the sensitivity of minor variant detection. Where single-site deletions resulted from sequencing errors, we edited the reads to match the T/F sequence. We excluded from analysis reads with multiple consecutive deletions or insertions from base calls out of phase with the flow order, and withheld from selection tests translations that contained premature stop codons. We used the R package ‘binom’ (version 1.0−5) to compute exact 95% confidence intervals from the binomial distribution, which quantifies uncertainty of variant frequencies due to resampling.

Bulk PCR primers:

1.R3.B3R (5′-ACTACTTGAAGCACTCAAGGCAAGCTTTATTG-3′; nt 9642-9611 HXB2).

BKB3F2 (5′ CGGGTTTATTACAGGGACAGCAG 3′; nt 4899–4921 HXB2)

454 patient-specific primer pairs for two amplicons (A & B):

A–F: GTGGGTCACAGTCTATTATGGG HXB2 nt 6326–6347

A–R: GGCTCAAAGGATACCTTTGGAC HXB2 nt 6859-6838

B–F: GGGATCAAAGCTTAAAACCATG HXB2 nt 6015–6036

B–R: GCATTGTCACTGAAATTGACTG HXB2 nt 6522-6501

Specific adapter sequences ligated to 5′ end of each primer for directional sequencing:

F: CGTATCGCCTCCCTCGCGCCATCAG

R: CTATGCGCCTTGCCAGCCCGCTCAG

### Virus replication fitness assays

Viral replication was assessed in activated primary CD4+ cells from normal human donors as previously described [8] with modifications. Relative replication rates were evaluated in parallel cultures infected by single virus strains and in competition cultures where cells were inoculated with identical numbers of two or three genetically-distinct virus strains. Relative growth rates were distinguished by PASS analysis. Fresh or frozen cells were treated with either 50 ng/ml or 3 µg/ml of staphylococcal enterotoxin B (Toxin Technology, Sarasota, FL) for 72 hours at 37°C to activate lymphocytes. 5×10^5^ cells were incubated with 50,000 IU of virus (multiplicity of infection 0.1) overnight at 37°C in 250 µl RPMI 1640 with 15% FBS and 30 U/ml IL-2. Cells were washed three times and plated in 24-well polystyrene tissue culture plates in a volume of 500 µl RPMI 1640 with 15% FBS and 30 U IL-2/ml. 50 µl of media was removed for day 1 p24 baseline analysis. Every 2 days, 50 µl lf media was removed and frozen for p24 analysis. For viral replication competition assays, cells were isolated and activated as described above. 1×10^6^ cells were incubated with 50,000 IU of each virus (for a combined multiplicity of infection of 0.1) overnight at 37°C in 250 µl RPMI 1640 with 15% FBS and 30 U/ml IL-2. Cells were washed three times and plated in 24-well polystyrene tissue culture plates in a volume of 1 ml RPMI 1640 with 15% FBS and 30 U IL-2/ml. 50 µl of media was removed for day 1 p24 baseline analysis, and 80 µl for an estimate of the input stock for PASS (see above). Every 2 days, 50 µl lf media was removed and frozen for p24 analysis and 80 µl was frozen for PASS analysis.

### Mathematical models for virus escape from Nab response

To investigate quantitative relationship between virus replication, diversification, and antibody-mediated selection, we extended a previous model of virus dynamics in HIV-1 infection [77]. In the new model, wild-type (WT) virus, 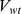, is defined as virus that has the T/F sequence in the Nab epitope under consideration. It is produced from infected cells, 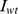, at rate 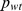 per cell and is cleared at rate 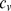 per virion. The virus infects uninfected target cells, 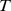, at rate 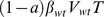, where 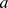 is the efficacy of circulating antibodies at neutralizing the wild-type virus and 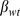 is the rate constant characterizing infection by WT virus in the absence of Nabs. A Nab escape mutant, 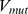, is resistant to neutralization by these Nabs. It infects target cells at rate 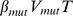, it is produced from infected cells, 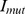, at rate 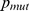 and it is cleared at rate 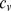 per virion. Cells producing virus die at rate 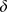. Uninfected target cells are produced at rate 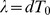 and die at rate 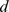. The dynamics of the virus and cell populations are thus given by the following equations:
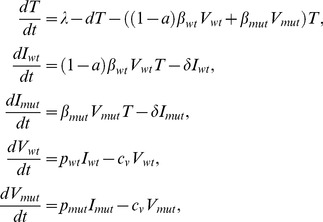
(1)where we assume that at the start of selection, both WT and escape variants are present in the population. Our results are quantitatively similar if we include generation of the escape variant from the T/F virus by mutation (not shown). Modeling escape of HIV from CTL responses, we and others have previously shown that the model (1) can be simplified due to the rapid clearance of viral particles from circulation [64] so as to consider only the dynamics of cells productively infected with wild-type virus, 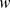, and escape mutant, 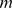. In this model the concentration of virus is directly proportional to the density of infected cells, so that the ratio 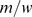 is also the ratio of mutant to WT virus. The simplified model is
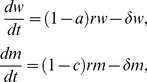
(2)where 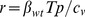 is the rate of growth of the population of cells infected with WT virus in the absence of a Nab response, and 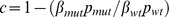 is the fitness cost of the escape mutation. Because the density of infected cells is proportional to the density of free virus particles, the rate of expansion of infected cells and free virus are identical. It is important to note that if target cell levels vary 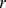 will be a function of time 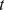. In the model (2) the dynamics of the ratio of the density of the escape variant to the WT virus in the population, 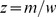, is given by 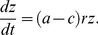
(3)


The change in frequency of the WT, or T/F virus in the population, 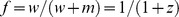 over time since the Nab response began is given by
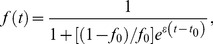
(4)where 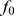 is the frequency of the WT virus at the start of Nab response, assumed to occur at time 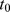, and 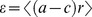 is the average rate of escape of the virus from Nab response over the considered time period [64]. It is clear from this model that escape will only occur if the efficacy of the Nab response *in vivo* is larger than the fitness cost associated with escape, 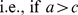.

The efficacy of the Nab response may change over time, for example, because of an increase in the level of Nabs. To describe the kinetics of the Nab response to the WT virus, we use a simple model where the level of Nabs begins increasing after a time delay, 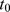, and saturates over time:
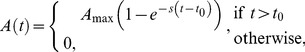
(5)where 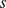 is the rate of increase of Nab levels over time. The efficacy of Nabs at blocking new infections is likely to be proportional to their concentration. We describe the change in Nab efficacy at blocking *de novo* infections by the Emax model commonly used in pharmacodynamic modeling, i.e.
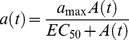
(6)where 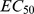 is the antibody concentration which is 50% neutralizing. By using eqns. (6) and (7) and numerically solving eqn. (3), we find the change in the frequency of the founder virus due to escape from the Nab response. In our SGA data the frequency of the WT or T/F virus sequence changes from 100% at an early time point to 0% at the subsequent time points. To estimate the minimal escape rate we replaced the value for the frequency of WT virus at the early time point, 100%, with 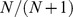 and at a later time point, 0%, with 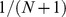 where 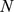 is the number of SGA-derived sequences available. This generates a lower bound estimate of the escape rate.

### Structural models of Nab escape mutations on CH40, CH77 and CH58 Env trimers

A model of HIV-1 gp120 for all regions except V1/V2 was constructed from crystal structures of HIV-1 gp120 core with complete N and C termini [48] and of HIV-1 gp120 core with V3 [46]. A model of V1/V2, meanwhile, was utilized directly from the scaffold determined context with PG9 [49]. The GlyProt server was used to model basic glycans at accessible potential N-linked glycosylation sites. Residues of gp120 involved in viral escape were mapped onto these atomic-level models. For the oligomeric viral spike context, the approximate locations of these residues were mapped as determined by cryo-electron microscopy [50]–[52].

## Supporting Information

Dataset S1 **Fitness costs of Nab escape mutations and mathematical modeling of Nab efficacy.**
(DOCX)

Figure S1 **Kinetics of autologous Nab responses, plasma viral load and CD4+ T lymphocyte counts.** Autologous plasma Nab titers against the respective T/F Envs for subjects CH40, CH77 and CH58, as measured by TZM assay and expressed as reciprocal IC_50_s, are shown in bold lines. Each value is the average of four independent experiments, performed in duplicate. The minimal plasma dilution used in the assay was 1∶20, and for this plot Nab titers of <1∶20 are denoted as zero. Each subject's plasma viral load is color-coded and represented by dashed lines; the CD4+T lymphocyte count is represented in black with the scale to the right.
(EPS)

Figure S2 **Replicative fitness of Nab escape mutants.** A. Replication kinetics of CH40 T/F, 6 mo, and 6 mo-Nab IMCs cultured in parallel. Virus stocks were generated on CD4+ T cells and equal M.O.I.s were inoculated onto parallel cultures of CD4+T cells from two seronegative donors. Viral p24 antigen was measured every 2 days. B. Replication kinetics of CH40 IMCs grown in direct competition. Equal quantities of viruses derived from CH40 6 mo IMC and CH40 6 mo-Nab IMC were co-cultured in the same CD4+ T lymphocytes. 50 ul of supernatant was removed every two days and assayed for viral growth by p24 antigen ELISA and tested for relative proportions by PASS. Replication kinetics of CH77 (C) and CH58 (D) T/F, 6 mo, and 6 mo-Nab IMCs. Results represent mean (+/− SD) of three independently performed experiments.
(EPS)

Figure S3
**Model of HIV infection and Nab escape in acute infection.** In this model, wild-type (WT) or T/F virus, 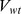, is produced from infected cells, 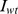, at rate 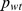 and is cleared at rate 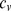 per virion. The virus infects uninfected target cells, 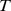, at rate 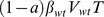 where 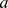 is the efficacy of circulating antibodies at neutralizing the wild-type virus. A Nab escape mutant, 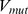, is resistant to neutralization by these Nabs. It infects target cells at rate 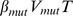, it is produced from infected cells, 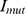, at rate 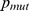, and it is cleared at the rate 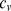 per virion. Cells producing virus die at the rate 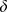, while uninfected target cells are produced at the rate 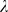 and die at the rate 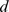.(EPS)

Figure S4
**Estimation of Nab efficacy to block** ***de novo*** **infections.** Using the data on the loss of the T/F sequence, we estimate the average efficacy of the Nab response at blocking *de novo* infections by the T/F virus. Our model assumed that the Nab response started at 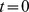 and had a constant average efficacy 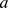. The rate of the loss of the founder virus 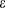 was directly proportional to the average *in vivo* efficacy of the Nab response, 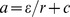, where 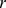 is the average rate of virus replication and 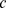 is the cost of escape mutations. The estimated escape rate was 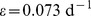 and 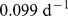 for CH40 and CH77 (SGA, panel A) and 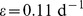 and 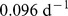 for CH40 and CH77 (PASS, panel B), respectively. Model predictions are shown in (A) and (B) by lines. The estimates of the average Nab efficacy were independent of the time at which Nab response started as long as that time was earlier than 45 (CH40) and 50 (CH77) days post-seroconversion. To determine whether the rise in Nab titers can predict viral escape, we estimated the kinetics of the Nab response from the experimental titer data using Eqn. (6) (panel C), and predicted the *in vivo* efficacy of the Nab response (panel D) and the loss of the T/F virus (panel E) using Eqns. (4) and (7). The estimated parameters for the kinetics of the Nab response are 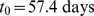 and 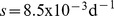 for CH40 and 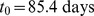 and 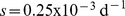 for CH77. The estimated half-saturation constant for Nab efficacy assuming 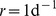 and 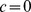 is 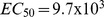 and 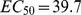 for CH40 and CH77, respectively.(EPS)

Table S1 **Frequency (%) of CH40 V1 Nab epitope sequence variants measured by SGA and PASS.**
(DOCX)

Table S2 **Frequency (%) of CH77 V2 Nab epitope sequence variants measured by SGA and PASS.**
(DOCX)
