# Supplementary material for: Early Low-Titer Neutralizing Antibodies Impede HIV-1 Replication and Select for Virus Escape
Source: PLoS Pathog. 2012 May 31;8(5):e1002721. doi: 10.1371/journal.ppat.1002721 (PMC3364956; doi:10.1371/journal.ppat.1002721)
Supplement: Dataset S1 — Fitness costs of Nab escape mutations and mathematical modeling of Nab efficacy. (DOCX) [file ppat.1002721.s001.docx]

**Dataset S1. Fitness costs of Nab escape mutations and mathematical modeling of Nab efficacy.**

Although several different amino acid combinations resulted in Nab resistance of quasispecies variants at the 6 month time point of CH40, two consensus amino acid substitutions, E146G and R327K, were present in the majority of the 14 SGA-derived genomes (Figure 2). The substantial effects of these two amino acid substitutions alone on neutralization susceptibility is illustrated in Figure 1B. To examine the fitness costs attributable to these two confirmed Nab escape mutations, we compared the replication rates of IMCs corresponding to the T/F sequence, the consensus 6 month sequence (6mo IMC) that contains both CTL and Nab escape mutations, and the consensus 6 month sequence excluding the two Nab mutations (6mo-Nab IMC). We performed these assays under a variety of *in vitro* culture conditions, including with PBMC and 293T derived virus stocks tested on primary activated CD4+ T lymphocyte cell cultures from different donors and with different cell stimulation protocols (see methods). For all variations of the experiments, we observed a reproducible order of replication fitness: the T/F virus IMC consistently replicated most efficiently, followed by the 6mo-Nab IMC (lacking Nab escape mutations), followed by the 6 mo IMC (containing Nab escape mutations) (Figure S2). We also conducted mixed-competition experiments between the 6mo IMC and the 6 mo-Nab IMC in the same cell cultures, and we quantified relative virus growth rates using PASS to distinguish the two IMC lineages. In the virus stock used to infect cells, the 6mo IMC represented a slightly higher percentage of total virus than the 6mo-Nab IMC (58% vs 42%). By day 4, the virus culture was in exponential growth and the 6mo-Nab variant had expanded to comprise greater than 60% of total virus; by day 8 the 6mo-Nab IMC expanded to 97% (Figure S2). Assuming unsynchronized virus replication with an average of 4 virus generations over 8 days [[3](#_ENREF_3)], we can estimate the minimum fitness cost of the Nab escape variant, *c*, with the equation: *c* = log(z(t)/z(0))/(r*t) (see methods). The result indicates a minimum fitness cost for the CH40 Nab escape variant of 24%. The estimated fitness cost would be higher if the replication rate is lower or if replication occurs over a shorter period of time (i.e. if replication starts later than t=0). Similar comparisons were conducted with CH77 and CH58 (Figure S2). We found no differences in replication rates between viruses with and without Nab escape mutations for CH77 and an approximately 10% difference for CH58.

To investigate how the kinetics of viral escape from the Nab response could inform our understanding of the efficacy of Nabs at blocking *de novo* infection, we extended the standard model of virus dynamics [[77](#_ENREF_77)] to encompass the early Nab response and viral escape from it. The model predicts the rate of accumulation of a given escape variant (and the associated loss of the T/F sequence) as the function of a number of parameters including the *in vivo* neutralizing efficacy of the Nab response, $a$, the speed of the generation of the Nab response, $s$, and the fitness cost of viral escape, $c$ (Figure S3). First, we used the kinetics of viral escape from Nabs to estimate the *in vivo* efficacy of the Nab response at blocking *de novo* infections. From the model [eqn. (4)] we estimated the rate at which the T/F sequence neutralizing epitope allele is lost in the viral population over time. Because sequencing could only be performed on available time points, only minimal estimates of the rate of viral escape could be calculated. The estimated escape rate is $\varepsilon=0.073 \text{day}^{-1}$ and $0.099\text{ day}^{-1}$ for CH40 and CH77 (based on SGA data, Figure S4A) and $\varepsilon=0.11 \text{day}^{-1}$ and $0.096 \text{day}^{-1}$ for CH40 and CH77 (based on PASS data, Figure S4B), respectively. The model suggests that the rate at which the T/F Nab epitope allele is lost is directly proportional to the average Nab efficacy, the associated fitness cost of Nab mutations, and the average rate of virus replication during the time period of viral escape.

The estimates of the average Nab efficacy are dependent on the average rate of virus replication over the time period in which Nab escape took place. In our two subjects, the loss of the T/F sequence due to escape from the Nab response occurred during the decline after peak viremia suggesting that virus replication might be minimal during this time period. A previous study comparing the rate of virus decline in the presence and absence of antiretroviral therapy during acute HIV infection found little evidence for virus replication [[9](#_ENREF_9)]. In a recent study that tracked the kinetics of viral escape from CTL responses during the decline from peak viremia, we found evidence that virus could be replicating at the rate 0.4 day^-1^ or higher in some patients [[38](#_ENREF_38)]. Because the maximal rate of HIV replication is likely to be in the order of 1-2 per day [[73](#_ENREF_73)], we used the replication rate $r=1 \text{day}^{-1}$for our analyses. The corresponding estimates of the average *in vivo* Nab efficacy, $<a>$, are therefore likely to be lower bound estimates, since according to Eq. (5), the Nab efficacy is equal to the escape rate divided by the average replication rate plus the attributable fitness cost, or $<a>=\frac{\varepsilon}{<r>}+c$.

Because the cost of escape, *c*, is not known for each escape mutation in each individual and has generally been believed to be low in the Env variable regions, we first set $c=0$ to obtain a lower bound on the *in vivo* Nab efficacy and then imputed the lower bound of our estimated fitness cost. In our model, the fitness cost simply adds to the estimated escape rate divided by the average viral replication rate, such that a fitness cost of 10% increases the estimated Nab efficacy by 10%. Calculated with $r=1 \text{day}^{-1}$ and fitness cost $c=0,$ the average *in vivo* efficacy of the Nab response specific to T/F virus as estimated from SGA data is $a=$7.3% and 9.9% in CH40 and CH77, respectively, and $a=$11.2% and 9.6% using PASS data for CH40 and CH77, respectively. Using the rate of replication *in vivo*, $r=1 \text{day}^{-1}$, and the minimum fitness cost of Nab escape mutations in CH40 is 24%, then the estimated average impairment to virus entry due to early strain-specific Nabs in CH40 is between 31.3% and 37.2%. If $r=0.4 \text{day}^{-1}$, as was shown recently by Fischer and colleagues based on the rapidity of accumulation of CTL escape variants during this time period [[38](#_ENREF_38)], then the minimum estimated Nab efficacy is between 42.3% and 48.8%. Thus, we estimate that in CH40, CH77 and CH58, the minimum costs to viral fitness of Nab escape mutations ranged from 0 to 24%, and the minimum estimates of Nab efficacy in impeding virus entry *in vivo* to be as much as 49% per replication cycle. Importantly, because of the rapid turnover of the virus and productively infected cells even a small *in vivo* efficacy of the Nab response ($\leq10\%$) is sufficient to cause viral escape in a matter a few weeks.

Next, we investigated whether the dynamics of the rising Nab titers (as measured *in vitro* by the TZM assay) can predict the kinetics of the loss of the T/F viral sequence. We estimated the rate of accumulation of Nabs and maximum Nab titers by fitting a simple model [eqn. (5)] to the experimental data (Figure S4C). The kinetics of the Nab response were then used to estimate changes in the *in vivo* efficacy of the Nab response assuming a standard Emax model from pharmacodynamics for the relationship between Nab concentration and neutralizing efficacy (eqn. (7), Figure S4D). Using this time-varying estimate of the Nab efficacy we predicted the time course of loss of the T/F sequence due to viral escape from the Nab response and estimated the half-saturation constant in the Emax model (Figure S4E). In subject CH40, Nab titers rose rapidly early in infection, thus a moderate increase in Nab efficacy was sufficient to cause the loss of T/F sequence over a period of 5 weeks. In CH40, the time-dependent Nab efficacy correlated well with the estimate of the average Nab efficacy obtained above using SGA/PASS data. In contrast, the Nab response in subject CH77 was delayed and of low magnitude (Figure S4C), necessitating a rapid increase in the predicted efficacy of Nab (Figure S4D) to cause the replacement of the T/F sequence in just two weeks (Figure S4E). Thus in CH77, neglecting the kinetics of the Nab response leads to a significant underestimation of the *in vivo* blocking efficacy of Nabs. Taken together, our analysis demonstrates that the kinetics of the Nab response is quantitatively consistent with the replacement of the T/F virus over the course of acute infection but the actual estimates of ${EC}_{50}$ and the *in vivo* efficacy of the Nab response depend strongly on the kinetics of the Nab response, the fitness cost of escape, and the rate of virus replication during the time of viral escape. The actual efficacy of early Nabs could be several fold higher if either a) the average replication rate of the T/F virus (in the absence of Nabs) during viral decline is lower than the assumed rate of$1 \text{d}^{-1}$; or b) there are greater fitness costs of mutations leading to viral escape; or c) Nabs accumulate more rapidly in a short time period; or d) if Nab efficacy fluctuates greatly over the first 120 days. Changing the model assumption of constant average Nab efficacy, imputing a higher viral replication rate, and explicitly taking into account the kinetics of Nabs in these patients leads to significantly higher estimates of the maximal efficacy of Nabs at blocking *de novo* infections (Figure S4 and unpublished data).
