# Supplementary material for: Early Low-Titer Neutralizing Antibodies Impede HIV-1 Replication and Select for Virus Escape
Source: PLoS Pathog. 2012 May 31;8(5):e1002721. doi: 10.1371/journal.ppat.1002721 (PMC3364956; doi:10.1371/journal.ppat.1002721)
Supplement: Table S1 — Frequency (%) of CH40 V1 Nab epitope sequence variants measured by SGA and PASS. (DOCX) [file ppat.1002721.s006.docx]

| TABLE S1. Frequency (%) of CH40 V1 Nab epitope sequence variants measured by SGA and PASS | | | | | | | | |
| --- | --- | --- | --- | --- | --- | --- | --- | --- |
| Sample date | Sequencing method | AA seq | EM (T/F seq) | KM | GM | TL | Other | Number of sequences |
|  |  | nt seq | GAA ATG (T/F seq) | AAA ATG | GGA ATG | ACA TTG |  |  |
| day 45 | SGA |  | 100 | 0 | 0 | 0 | 0 | 14 |
|  | PASS |  | 98.5 | 1.1 | 0 | 0 | 0.4 | 459 |
| day 111 | SGA |  | 13 | 75 | 13 | 0 | 0 | 8 |
|  | PASS |  | 4 | 87 | 9 | 0 | 0 | 127 |
| day 132 | SGA |  | 5 | 50 | 27 | 0 | 0 | 22 |
|  | PASS |  | 19 | 69 | 24 | 0 | <1 | 269 |
| day 181 | SGA |  | 14 | 0 | 71 | 0 | 0 | 7 |
|  | PASS |  | 12 | 16 | 69 | 0 | 3 | 771 |
| day 284 | SGA |  | 50 | 33 | 0 | 8 | 0 | 12 |
|  | PASS |  | 48 | 5 | 4 | 8 | 35 | 130 |
| day 412 | SGA |  | 0 | 0 | 8 | 92 | 0 | 12 |
|  | PASS |  | 1 | 0 | 19 | 78 | 2 | 1741 |
