# Supplementary material for: Early Low-Titer Neutralizing Antibodies Impede HIV-1 Replication and Select for Virus Escape
Source: PLoS Pathog. 2012 May 31;8(5):e1002721. doi: 10.1371/journal.ppat.1002721 (PMC3364956; doi:10.1371/journal.ppat.1002721)
Supplement: Table S2 — Frequency (%) of CH77 V2 Nab epitope sequence variants measured by SGA and PASS. (DOCX) [file ppat.1002721.s007.docx]

| TABLE S2. Frequency (%) of CH77 V2 Nab epitope sequence variants measured by SGA and PASS | | | | | | | | | | | | | |
| --- | --- | --- | --- | --- | --- | --- | --- | --- | --- | --- | --- | --- | --- |
| Sample date | Sequencing method | AA seq | KT  (T/F seq) | KN | KI | ET | KA | TT | KV | del^a^ | EN | Other | Total (#) |
|  |  | nt seq | AAA ACT (T/F seq) | AAA AAT | AAA ATT | GAA ACT | AAA GCT | ACA ACT | AAA GTT | ... ... | GAA AAT |  |  |
| day 32 | SGA |  | 100 | 0 | 0 | 0 | 0 | 0 | 0 | 0 | 0 | 0 | 12 |
|  | PASS |  | 99.8 | 0.2 | 0 | 0 | 0 | 0 | 0 | 0 | 0 | 0 | 468 |
| day 102 | SGA |  | 46 | 8 | 0 | 8 | 8 | 15 | 0 | 0 | 0 | 8 | 13 |
|  | PASS |  | 33 | 11 | 13 | 11 | 11 | 7 | 4 | 2 | 0 | 7 | 46 |
| day 159 | SGA |  | 0 | 41 | 0 | 7 | 7 | 7 | 0 | 41 | 0 | 0 | 17 |
|  | PASS |  | 4 | 45 | 0 | 6 | 2 | 0 | 0 | 41 | 0 | 3 | 312 |
| day 592 | SGA |  | 0 | 20 | 0 | 0 | 0 | 0 | 0 | 0 | 80 | 0 | 5 |
|  | PASS |  | 0 | 3 | 0 | 0 | 0 | 0 | 0 | 0 | 91 | 5 | 811 |

^a^Indicates two amino acid/six nucleotide deletion.
